# JNK3 regulates β cell responses to incretins in human islets and mouse models

**DOI:** 10.1172/JCI185707

**Published:** 2026-01-02

**Authors:** Ruy A. Louzada, Marel Gonzalez Medina, Valentina Pita-Grisanti, Jessica Bouviere, Amanda F. Neves, Joana Almaça, Myoung Sook Han, Roger J. Davis, Gil Leibowitz, Manuel Blandino-Rosano, Ernesto Bernal-Mizrachi

**Affiliations:** 1 Division of Endocrinology, Diabetes, and Metabolism, Department of Medicine and; 2 Department of Biochemistry and Molecular Biology, University of Miami Miller School of Medicine, Miami, Florida.; 3 Program in Molecular Medicine, University of Massachusetts Chan Medical School, Worcester, Massachusetts, USA.; 4 Diabetes Unit and Endocrine Service, Hadassah-Hebrew University Medical Center, Jerusalem, Israel.; 5 Veterans Affairs Medical Center, Miami, Florida, USA.

**Keywords:** Endocrinology, Metabolism, Apoptosis, Beta cells, Insulin

## Abstract

The c-Jun N-terminal kinases (JNKs) regulate diverse physiological processes. Whereas JNK1 and JNK2 are broadly expressed and associated with insulin resistance, inflammation, and stress responses, JNK3 is largely restricted to central nervous system neurons and pancreatic β cells, and its physiological role in β cells remains poorly defined. To investigate its function, we generated mice lacking JNK3 specifically in β cells (βJNK3-KO). These mice displayed glucose intolerance and defective insulin secretion, particularly after oral glucose challenge, indicating impaired incretin responses. Consistently, Exendin-4–stimulated (Ex4-stimulated) insulin secretion was blunted in βJNK3-KO islets, accompanied by reduced GLP-1R expression. Similar findings were observed in human islets treated with a selective JNK3 inhibitor (iJNK3). Downstream of GLP-1R, Ex4-induced CREB phosphorylation was diminished in βJNK3-KO islets, indicating impaired canonical signaling. Moreover, activation of the GLP-1R/CREB/IRS2 pathway, a key regulator of β cell survival, was reduced in βJNK3-KO islets and iJNK3-treated human islets. As a consequence, the protective effects of Ex4 were lost in cytokine-treated βJNK3-KO and human islets, and Ex4-mediated protection was partially attenuated in βJNK3-KO mice exposed to multiple low-dose streptozotocin. These findings identify JNK3 as a regulator of β cell function and survival and suggest that targeting this pathway may enhance incretin-based therapies.

## Abstract 2

JNK3 is crucial for β-cell function and survival, influencing incretin response and insulin secretion, making it a potential therapeutic target for diabetes.

## Introduction

Type 1 diabetes (T1D) and type 2 diabetes (T2D) are characterized by the autoimmune destruction of pancreatic β cells and by defective β cell adaptation to insulin resistance, respectively. Cellular stress is a common path for altered β cell homeostasis in these diseases. In T1D, infiltration of inflammatory cells and proinflammatory cytokines (CTKs) drive β cell death (1). The c-Jun amino-terminal kinase (JNK) pathway, also known as stress-activated protein kinases (SAPKs) pathway, is activated by endoplasmic reticulum and oxidative stress and has been implicated in cytokine-mediated β cell apoptosis (2). Three genes encode JNK isoforms: JNK1 (Mapk8), JNK2 (Mapk9), and JNK3 (Mapk10), with alternative splicing yielding 10 protein sequences with significant homology (3). While JNK1 and JNK2 are ubiquitously expressed, including in pancreatic β cells, and play a role in survival, JNK3 is predominantly found in neurons, testes, and pancreatic islets (4), and its role has been less explored.

Evidence supports the involvement of JNK1 and JNK2 in various diabetic models, including those mimicking T1D using streptozotocin (STZ), nonobese diabetic mice, and proinflammatory cytokines to induce apoptosis. The finding that deletion of JNK1 prevented the impairment of insulin secretion and β cell survival induced by proinflammatory cytokines (2, 5, 6) suggests that targeting JNK1 and JNK2 in β cells could be a new therapeutic approach for diabetes (7–9). Pharmacological inhibition of JNK and knockout animal models of JNK1 and JNK2 have shown enhanced islet survival under transplantation and diabetogenic conditions (2, 10). In contrast, overactivation of JNKs in a transgenic mouse model overexpressing MKK7 in β cells resulted in insulin resistance and impaired glucose homeostasis in β cells, despite the absence of abnormal morphology or caspase 3 activation (11). This suggests a potential redundant and compensatory mechanism among the 3 JNK isoforms in β cells. However, the specific contribution of JNK3 to diabetes pathophysiology remains unclear.

While proapoptotic signals in β cells converge on the JNK pathway, survival mechanisms such as the cAMP/CREB/IRS2/AKT pathway counteract the fate of pancreatic β cells under proinflammatory cytokine exposure (12–15). Intriguingly, JNK3 deletion in mouse and human β cell lines increased susceptibility to death upon proinflammatory cytokine exposure (4), suggesting an opposite effect of JNK3 compared with JNK1 and JNK2 in apoptosis. Additionally, JNK3 silencing in insulinoma cells markedly decreased insulin receptor substrate 2 (IRS2) expression (16). Finally, JNK3 signaling by dual leucine zipper-bearing kinase (Dlk, MAP3K12) was shown to be a key mechanism in adapting islet β cell mass during postnatal development and weight gain (17). This evidence suggests that JNK3 has protective effects in in vitro models, but there is a gap in knowledge that centers on the role of β cell JNK3 in controlling glucose homeostasis and β cell mass in vivo.

Here, we generated an animal model to delete JNK3 specifically in β cells and observed an impairment of the incretin response in vivo. Mechanistically, GLP-1R expression and canonical signaling were decreased in mouse and human islets deficient in JNK3, rendering these islets more susceptible to apoptosis induced by proinflammatory cytokines. Furthermore, the beneficial effect of GLP-1R agonist treatment in a preclinical model of diabetes was partially attenuated in JNK3-deficient mice. Given that GLP-1R agonists are crucial medications for treating diabetes, identifying the signaling pathways and mechanisms involved in the response to GLP-1R agonists could have significant implications for diabetes therapy. These studies support a role for JNK3 in in vivo incretin responses in β cells and suggest that this pathway can be used to enhance therapeutic actions of GLP-1R agonists.

## Results

### JNK3 mRNA is the most abundant JNK isoform in β cells and is induced in human diabetes.

First, we assessed mRNA expression of JNK1, JNK2, and JNK3 in the single-cell transcriptome of α and β cells from mice at different developmental and postnatal stages (18). This shows that, while JNK1 and JNK2 decrease during the first 60 days of postnatal life, JNK3 mRNA is low during development and increases after postnatal day 3 to day 60 (Supplemental Figure 1A; supplemental material available online with this article; https://doi.org/10.1172/JCI185707DS1). Analysis of JNK1, JNK2, and JNK3 mRNA in the single-cell transcriptome library from the human pancreas (19) showed that JNK1 and JNK2 mRNA were present in all islet cells and acinar and ductal compartments (Supplemental Figure 2A). In contrast, JNK3 mRNA was more abundant in human α and β cells, while it was barely detected in exocrine and ductal cells (Supplemental Figure 2A). Moreover, the JNK3 isoform was found to be the most abundant isoform in β cells (Supplemental Figure 2A). Analyzing data from 65 HPAP donors (20), we confirmed the expression of JNK1, JNK2, and JNK3 across different pancreatic cell types. Notably, JNK3 expression was increased in mice subjected to a high-fat diet (HFD) compared with a regular chow diet (21) (Supplemental Figure 1B) and the expression of all 3 isoforms was increased in pancreatic β cells from type 2 diabetic donors (Supplemental Figure 2A) and in Type 1 diabetes donors in a single-cell analysis (Supplemental Figure 3A), indicating the involvement of JNKs in β cell adaptation to diabetes.

### Deletion of JNK3 in β cells results in glucose intolerance and defective insulin secretion in response to oral glucose and incretin stimulation.

**Figure 1:**
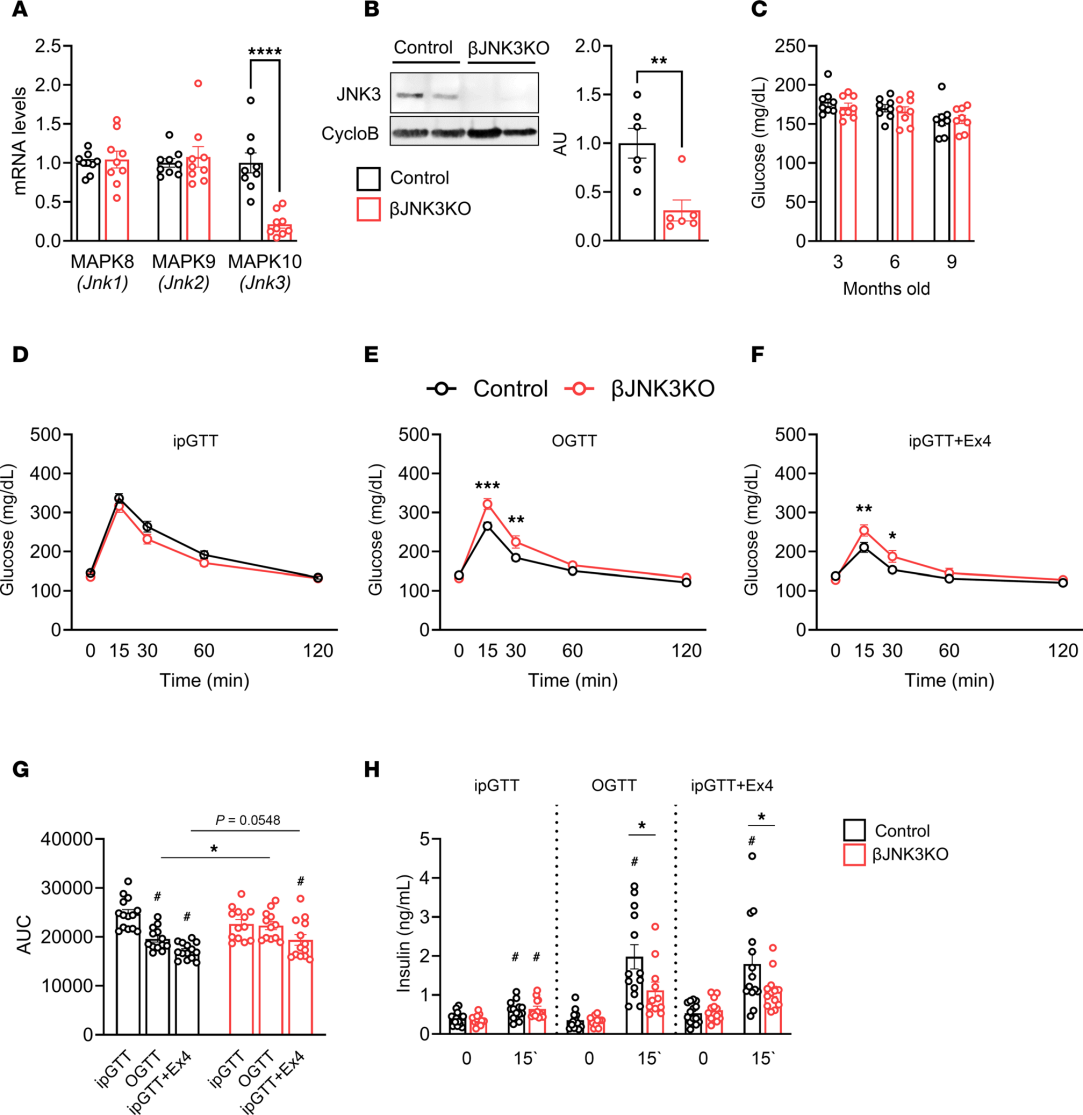
Deletion of JNK3 in β cells results in glucose intolerance and defective insulin secretion in response to oral glucose and incretin stimulation. (**A**) RT-PCR from 6-month-old male Control and βJNK3-KO islets. (**B**) Immunoblotting and quantification for JNK3 levels in isolated islets from 6-month-old male Control and βJNK3-KO. (**C**) Random fed glucose during the first 9 months of age. (**D**) Intraperitoneal glucose tolerance test (ipGTT), (**E**) Oral Glucose tolerance test (OGTT), (**F**) Coadministration of Exendin 4 during ipGTT in males of 4–5 months of age. (**G**) Calculation of the AUCs in all glucose tolerance tests and (**H**) Insulin levels at baseline and 15 minutes after glucose tolerance tests. Data are expressed as means ± SEM. Statistical significance was determined by 2-way ANOVA. *P < 0.05, **P < 0.01, ***P < 0.001, ***P < 0.0001 between groups; ^#^*P* < 0.05 within the same group.

To further assess the role of JNK3 in β cells in vivo, we generated mice with homozygous JNK3 deletion by crossing floxed-JNK3 mice with Rip-Cre (data not shown) and Ins-Cre mice (βJNK3-KO) (22–24). Islets isolated from βJNK3-KO mice showed a marked reduction in MAPK10 (JNK3) gene expression and JNK3 protein levels, without any compensatory effect on the levels of MAPK8 (JNK1) and MAPK9 (JNK2) mRNA (Figure 1, A and B). Random glucose levels in 3-, 6-, and 9-month-old βJNK3-KO mice were not different from controls (Figure 1C), as were the proportion of Ki67-positive β cells and overall β cell mass (Supplemental Figure 4, A and B, respectively). However, both male and female βJNK3-KO mice exhibited glucose intolerance (Figure 1, D and E) and impaired insulin secretion (Figure 1F) only during oral glucose tolerance test (OGTT), with no differences when glucose was administered intraperitoneally (Figure 1, D, E, and G, and Supplemental Figure 4, C–F for female βJNK3-KO mice). Further evaluation of the incretin effect by coadministering i.p. glucose with the GLP-1R agonist, Exendin 4 (Ex4), confirmed the impaired response to incretin signals (Figure 1F). This was accompanied by blunted insulin secretion (Figure 1H), suggesting a role for JNK3 in incretin induced insulin secretion in vivo.

### Inducible deletion of JNK3 in mature β cells results in glucose intolerance and defective insulin secretion after oral glucose and nutrients.

**Figure 2:**
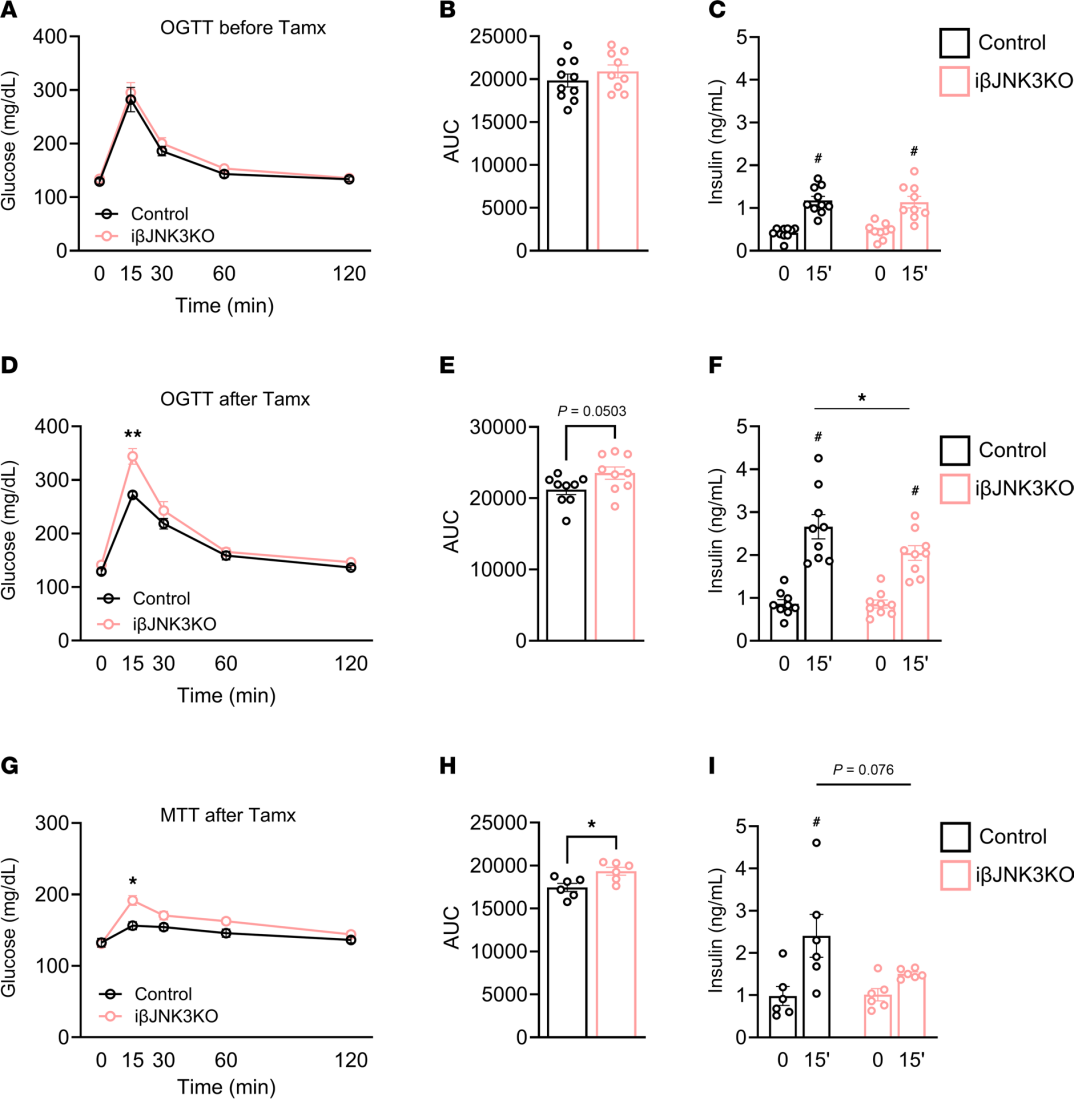
Inducible deletion of JNK3 in mature β cells results in glucose intolerance and defective insulin secretion after oral glucose and nutrients. (**A**) Oral glucose tolerance test, (**B**) AUC, and (**C**) insulin levels before Tmx injection in 4-month-old male mice. (**D**) Oral glucose tolerance test, (**E**) AUC, and (**F**) insulin levels at 5 weeks after Tmx injection. (**G**) Meal Tolerance Test, (**H**) AUC, and (**I**) insulin levels at 6 weeks after Tmx injection. Data are expressed as means ± SEM. Statistical significance was determined by 2-way ANOVA. *P < 0.05, **P < 0.01, ***P < 0.0001 between groups; ^#^*P* < 0.05 within the same group.

To eliminate potential effects arising from loss of JNK3 during developmental stages, we disrupted JNK3 in adult β cells by crossing floxed-JNK3 model with mice expressing a tamoxifen-inducible Cre under the control of the mouse Ins1 promoter (Mip-CreERTM), named iβJNK3-KO (22). JNK3 protein levels showed a marked reduction in the iβJNK3-KO (Supplemental Figure 5A). Before tamoxifen treatment, iβJNK3-KO mice were normoglycemic and exhibited normal glucose tolerance in both i.p. (data not shown) and oral glucose tolerance tests (Figure 2, A and B) and comparable insulin secretion in response to oral glucose (Figure 2C). However, 5 weeks after tamoxifen (Tmx) administration, iβJNK3-KO mice showed impaired glucose clearance and decreased insulin secretion after oral glucose administration (Figure 2, D–F). Further testing of incretin responses in vivo using meal tolerance test (MTT) (25) confirmed glucose intolerance and blunted insulin secretion 6 weeks after Tmx (Figure 2, G–I).

### JNK3 inhibition reduces in vitro GLP-1R–mediated signaling and insulin secretion.

**Figure 3:**
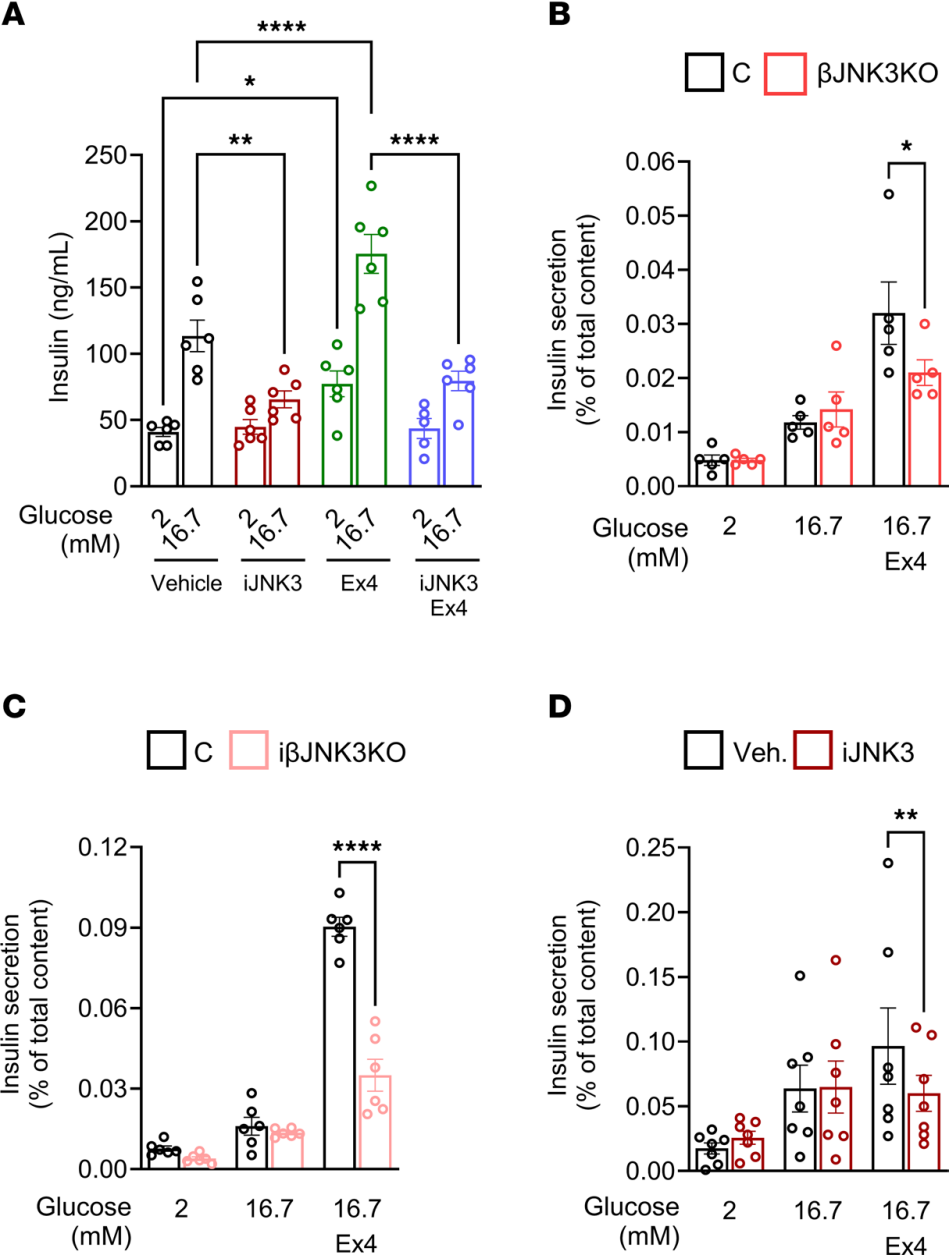
JNK3 inhibition reduces in vitro GLP-1R–mediated insulin secretion. (**A**) Assessment of insulin secretion in MIN6 induced by glucose, or combination of glucose with the GLP-1R agonist Exendin 4 (Ex4) in the presence or absence of a JNK3 inhibitor (iJNK3). Glucose-stimulated insulin secretion alone or in combination with Ex4 in (**B**) isolated islets from Control and βJNK3-KO mice, (**C**) isolated islets from Control and iβJNK3-KO,and (**D**) human islets from donors no. 4–6, 8–10, and 15 were treated with vehicle or iJNK3 for 24 hours. Data are expressed as means ± SEM. Statistical significance was determined by 2-way ANOVA. *P < 0.05, **P < 0.01, ***P < 0.001, ***P < 0.0001 between groups.

To gain insights into potential mechanisms by which JNK3 deletion impairs insulin secretory responses to incretins, we used MIN6 cells treated with iJNK3 (26). As expected, iJNK3 decreased the phosphorylation of c-JUN induced by CTKs, a downstream target of JNKs, compared with a broad-spectrum inhibitor of JNKs, SP600125 (Supplemental Figure 5B). Insulin secretory responses to glucose in MIN6 cells treated with iJNK3 were blunted in the presence or absence of Ex4 (Figure 3A). Although total insulin content did not differ between groups (Supplemental Figure 6, A–C), glucose-stimulated insulin secretion (GSIS) was decreased in βJNK3-KO and iβJNK3-KO after stimulation with a combination of high glucose and Ex4, but not after high glucose alone (Figure 3, B and C). Human islets treated with iJNK3 also exhibited a reduction in insulin secretion upon high glucose in combination with Ex4 treatment (Figure 3D).

**Figure 4:**
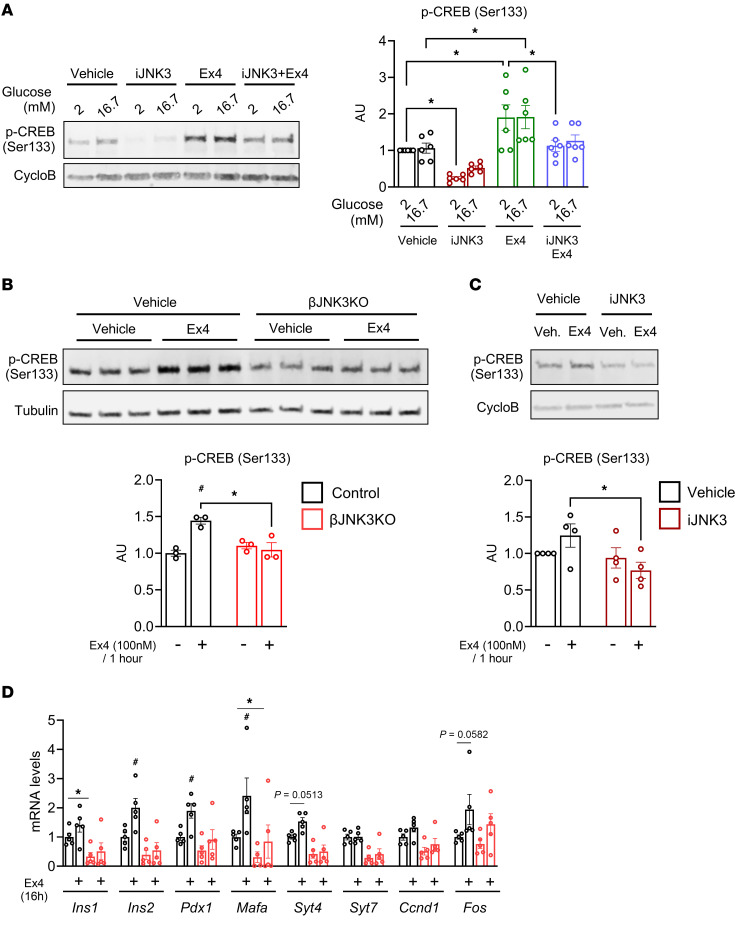
JNK3 inhibition reduces in vitro GLP-1R–mediated signaling by reduction of *GLP-1R* gene expression. (**A**) Phosphorylation of CREB in MIN6 cells after 1 hour of Exendin 4 treatment in the presence or absence of a JNK3 inhibitor (iJNK3). (**B**) Phosphorylation of CREB in isolated islets from 4–6 month-old Control and βJNK3-KO male and female mice after 1 hour of Exendin 4 treatment (**C**) Phosphorylation of CREB in human islets from donors no. 4–7 treated with vehicle or iJNK3 after 1 hour of Exendin 4 treatment. (**D**) RT-PCR for β cell maturity genes in isolated islets from control and βJNK3-KO treated with Ex4 for 16 hours. Data are expressed as means ± SEM. Statistical significance was determined by 2-way ANOVA. *P < 0.05 between groups; ^#^*P* < 0.05 within the same group.

Assessment of incretin signaling showed a decrease in PKA activity (Supplemental Figure 7A) and consequently CREB phosphorylation in MIN6 cells cultured in low glucose in the presence or absence of Ex4 treatment after iJNK3 treatment (Figure 4A). Lower PKA activity and CREB phosphorylation upon Ex4 treatment was also observed in βJNK3-KO islets (Supplemental Figure 7B and Figure 4B) and after pharmacological inhibition of JNK3 in human islets (Supplemental Figure 7C and Figure 4C). Next, we analyzed key β cell identity genes and CREB-responsive genes in mouse and human islets (Supplemental Figure 6, D and E, respectively). To determine whether the expression of these genes is affected by JNK3 inhibition, we measured mRNA levels after 16 hours of Ex4 treatment. In βJNK3-KO islets, the induction of key β cell and CREB-sensitive genes (27) was attenuated (Figure 4D). These studies demonstrate that induction of cAMP/PKA/CREB axis by GLP-1R agonist is mediated at least in part by JNK3.

To assess the cAMP/PKA/CREB axis downstream of GLP-1R activation, we performed Forskolin treatment. Insulin secretion induced by high glucose plus Forskolin was comparable between control and βJNK3-KO islets, as well as in human islets treated with iJNK3 (Supplemental Figure 8, A and D, respectively). Similarly, Forskolin induced CREB-responsive genes to the same extent in βJNK3-KO and control islets (Supplemental Figure 8C), indicating that downstream GLP-1R signaling remains intact in the absence of JNK3.

**Figure 5:**
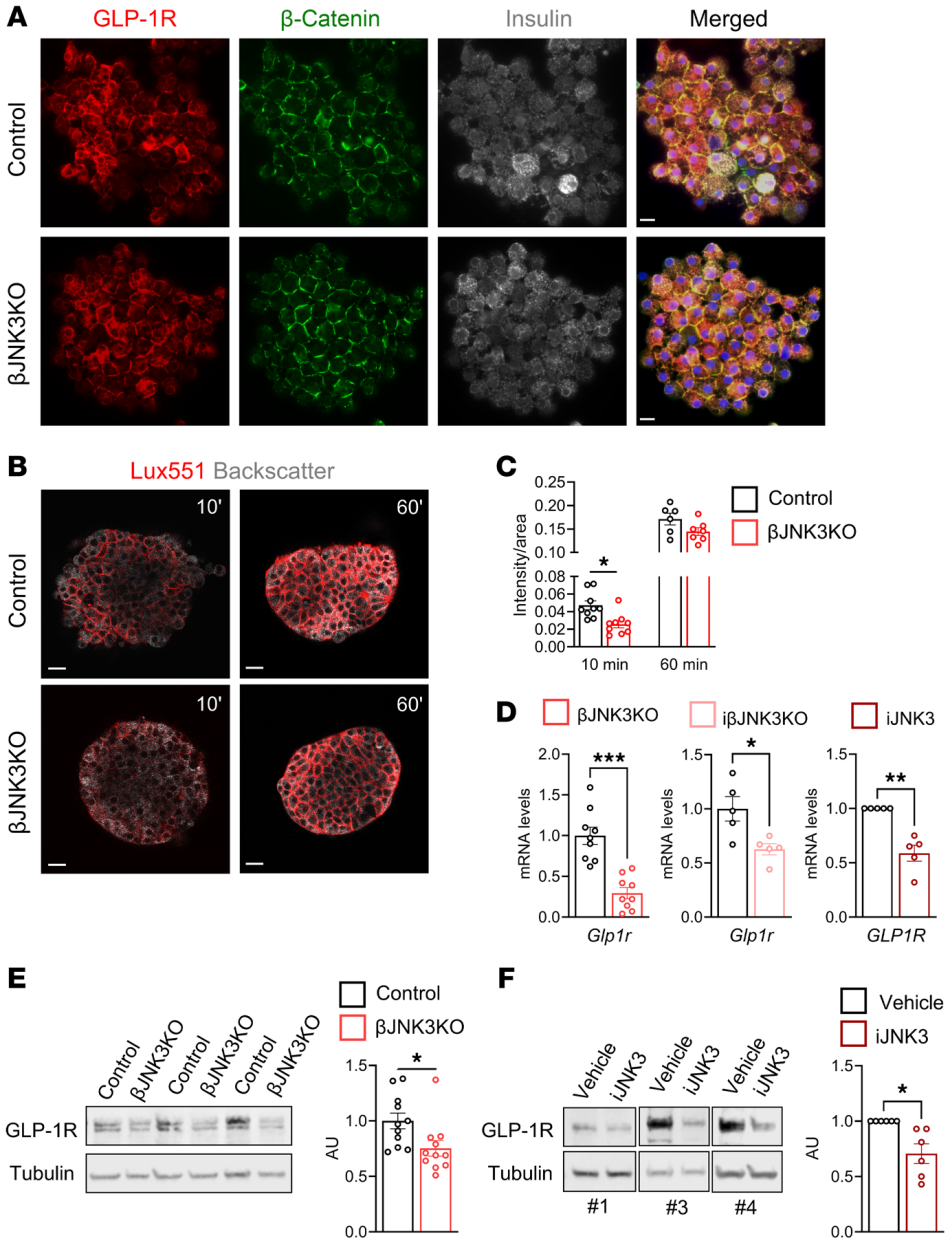
JNK3 inhibition reduces *GLP-1R* gene expression. (**A**) Representative images of dispersed islets GLP-1R (red) beta catenin (green) insulin (gray) and DAPI (blue); scale bar: 10 μm. (**B**) Representative images of live islets incubated with Luxendin551. Scale bar: 20 μm. (**C**) Quantification of Luxendin551 membrane labeling at 10 and 60 minutes. (**D**) q-RT-PCR analysis of *Glp1r* mRNA levels in isolated islets from (**E**) control and βJNK3-KO mice, control and iβJNK3-KO mice, and human islets treated with iJNK3 from donors 1–5, (**F**) GLP-1R protein levels in human islets from donors no. 1, 3–7 treated with vehicle or iJNK3 for 24 hours. Data are expressed as means ± SEM. Statistical significance was determined by 2-way ANOVA. *P < 0.05 between groups; ^#^*P* < 0.05 within the same group.

Given that JNK3 inhibition decreased induction of the cAMP/PKA/CREB axis induced by GLP-1R agonist, we next focused on proximal events involving GLP-1R localization and expression. GLP-1R was colocalized with β catenin at the plasma membrane of insulin-positive cells in βJNK3-KO islets, indicating that JNK3 deficiency does not affect GLP-1R trafficking or localization (Figure 5A). To quantitatively assess GLP-1R levels at the cell membrane, we labeled the receptor using Luxendin551 (28). After 10 minutes of incubation, clear membrane staining was observed in control islets but was reduced in βJNK3-KO islets. By 60 minutes, membrane staining was strong and comparable between groups (Figure 5, B and C), suggesting that reduced GLP-1R abundance may contribute to impaired incretin signaling in βJNK3-KO islets.

### JNK3 inhibition reduces GLP1R gene expression.

Bioinformatic analysis of the promoter region of the *GLP1R* gene in humans and mice showed enrichment of putative binding sites for transcription factors targeted by JNKs, such as cJUN, JUNB, Elk1, and other substrates (29) (Supplemental Figure 9). *Glp1r* mRNA levels were downregulated in both βJNK3-KO and iβJNK3-KO islets (Figure 5D), and this was accompanied by reduced GLP-1R protein levels in βJNK3-KO islets (Figure 5E). Single-cell RNA-seq analysis showed a positive correlation between *JNK3* and *GLP1R* expression (Supplemental Figure 2B) (19), as well as between *JNK2/JNK3* and *GLP1R* (Supplemental Figure 3B) (20). Similarly, human islets treated with iJNK3 displayed decreased *GLP1R* mRNA levels (Figure 5D) and protein expression (Figure 5F). Together, these findings indicate that JNK3 regulates incretin-mediated insulin secretion, at least in part, by controlling *GLP1R* expression.

**Figure 6:**
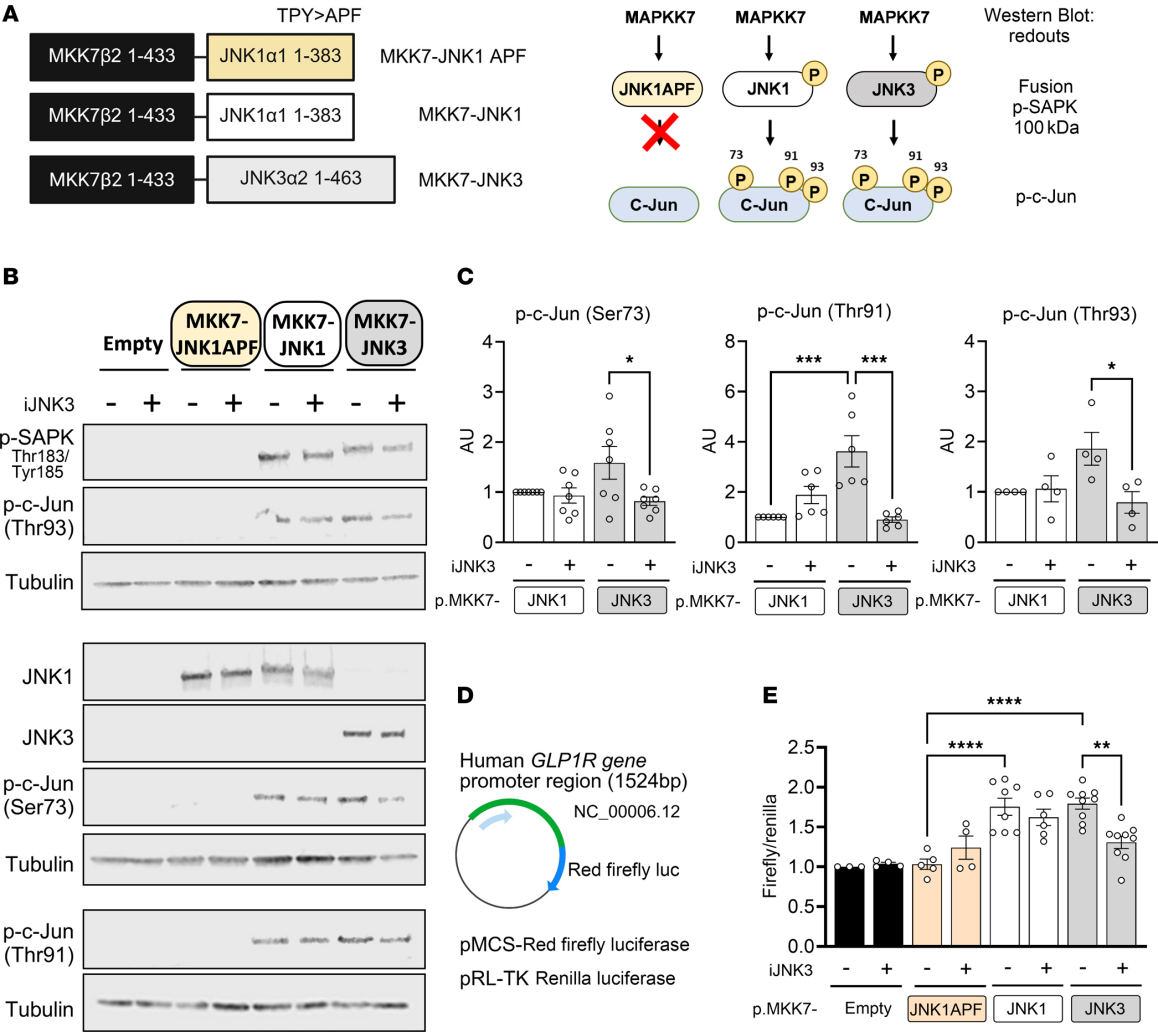
Transcriptional activation of the *GLP1R* is directly mediated by JNK. (**A**) Schematic representation of the MKK7-JNK fusion proteins. The constructs contain residues 1–443 of MKK7 fused to JNK1α1 (1–383), JNK3α2 (1–463), or phosphorylation-negative JNK (Thr180–Pro–Tyr182 replaced with Ala–Pro–Phe; APF). (**B**) Expression of MKK7-JNK fusion proteins in HEK293T cells detected by immunoblotting (IB). The presence of JNK1, JNK2, and JNK3 fusion proteins in cell lysates was confirmed. JNK phosphorylation status was assessed using an anti–phospho-SAPK antibody. (**C**) JNK activation was evaluated by IB using an antibody to phospho–c-Jun (Ser73, Thr91, and Thr93). (**D**) Schematic of the cloned promoter region of the human *GLP1R* (~1500 bp upstream to transcription start site) into the pMCS-Red Firefly luciferase vector. Renilla luciferase (pRL-TK) was used as an internal control. (**E**) Firefly/Renilla luciferase ratio indicating increased transcriptional activation of the human *GLP1R* promoter region. Data are expressed as means ± SEM. Statistical significance was determined by 2-way ANOVA. *P < 0.05 between groups; #*P* < 0.05 within the same group.

To determine whether JNK activation regulates GLP-1R expression, we used a plasmid encoding a constitutively active MKK7, the upstream JNK kinase, linked to JNK as a fusion protein, providing an in vitro strategy to selectively increase JNK activity (Figure 6A). The MKK7-JNK1APF (dominant-negative version of the JNK1 protein mutant) showed no JNK phosphorylation and, consequently, no c-JUN phosphorylation (Figure 6B). In contrast, MKK7-JNK1 and MKK7-JNK3 constructs induced the expression of the expected approximately 100 kDa fusion proteins, with both JNK1 and JNK3 phosphorylated, leading to increased phosphorylation of c-JUN at Ser73, Thr91, and Thr93. Notably, iJNK3 inhibited c-JUN phosphorylation at all sites, only in MKK7-JNK3–transfected cells, demonstrating specificity of this inhibitor (Figure 6C).

To test transcriptional regulation, the human *GLP1R* promoter region was cloned upstream of a luciferase reporter and cotransfected into HEK293T cells (Supplemental Figure 9 and Figure 6D). Both MKK7-JNK1 and MKK7-JNK3 increased the Firefly/Renilla luciferase ratio, indicating enhanced transcriptional activation of the *GLP1R* promoter. Importantly, iJNK3 specifically reduced the promoter activation induced by MKK7-JNK3 overexpression (Figure 6E). These results suggest the presence of JNK3-dependent transcriptional activation and functional binding sites within the approximately 1500 bp *GLP1R* promoter region.

### Overactivation of JNK3 did not potentiate incretin responses.

We first overexpressed JNK3 in control mouse islets using an adenoviral vector (Adenovirus-JNK3). GSIS experiments revealed similar responses to high glucose and Ex4 treatment compared with controls (Supplemental Figure 10, A and B). To further test whether increased JNK3 activity could potentiate incretin responses, MIN6 cells were transiently transfected with the constructs (Supplemental Figure 11A). Phosphorylation of CREB and insulin secretion were comparable between groups (Supplemental Figure 11, B and C). Although plasmid transfection efficiency is typically low in human islets, we dispersed human islets into single cells and performed transient transfection with the constitutively active JNK3 construct. While no quantitative data on transfection efficiency were obtained, JNK3 activation did not increase *GLP1R* or *IRS2* expression, nor did it enhance insulin secretion (Supplemental Figure 11, D and E).

### Beneficial effect of Ex4 in improving glucose homeostasis and preserving β cell mass after low-dose STZ is partially dependent on JNK3 in β cells.

**Figure 7:**
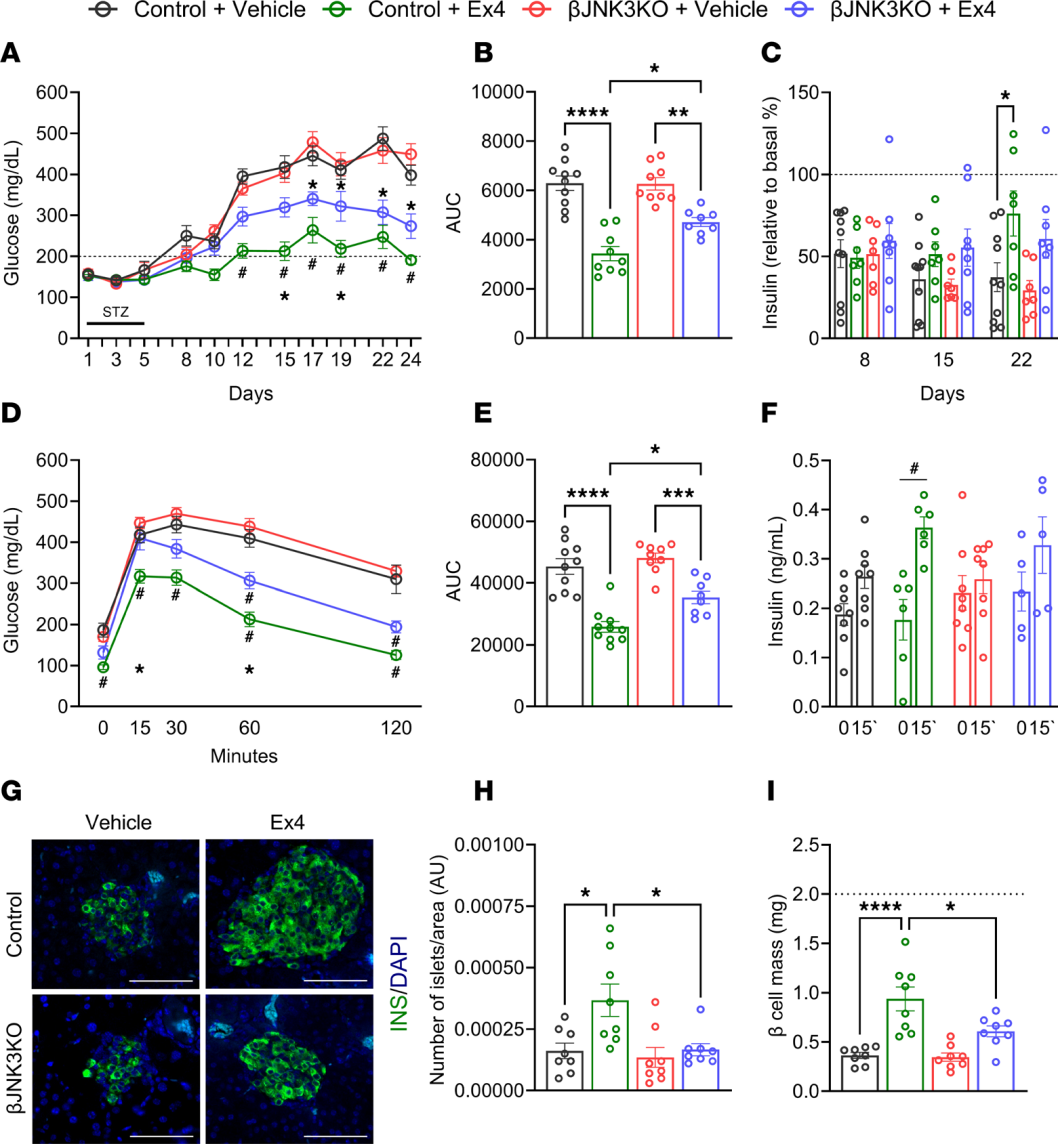
Beneficial effect of Ex4 in improving glucose homeostasis and preserving β cell mass after low-dose STZ is partially dependent on JNK3 in β cells. (**A**) The data represents the average glucose of 2 to 3 measurements per week. Control Vehicle group (Black), Control+Ex4 group (Green), βJNK3-KO+Vehicle group (Red), and βJNK3-KO+Ex4 group (Blue) (*n* = 9–10). Treatment started 2 days before STZ protocol and was conducted daily for 4 weeks. Ex4 was injected intraperitoneally at 1 nmol/kg. (**B**) AUC over 4 weeks after STZ protocol. (**C**) Insulin levels at days 8, 15, and 22, normalized to the baseline (dashed lines) of each animal before STZ protocol. (**D**) Meal tolerance test (MTT) at 15 days after STZ, (**E**) AUC, and (**F**) insulin levels during MTT baseline and 15 minutes after gavage. (**G**) Representative images of islets insulin (green) and DAPI (blue); scale bar: 75 μm, (**H**) number of islets per area and (**I**) β cell mass of pancreases from all the groups. Dashed lines in **I** represent average of normoglycemic mice, for reference. In panels **A** and **D**, *P < 0.05 indicates differences between groups (Control versus KO treated with Ex4), and #*P* < 0.05 indicates differences within groups (vehicle versus Ex4 treated within the same genotype). *P < 0.05, **P < 0.01, ***P < 0.001, ****P < 0.0001. The results are expressed as means ± SEM.

Given the role of JNK3 on insulin secretory responses induced by GLP-1R, we tested the effects of JNK3 on incretin-mediated β cell regeneration in the low-dose STZ mouse model. This model exhibits progressive inflammation followed by the destruction of β cells (13, 30). Daily administration of Ex4 (1 nmol/kg/d) or vehicle control intraperitoneally starting 2 days before treatment with low-dose STZ (5 consecutive injections of 40 mg/kg/d of STZ) resulted in comparable glucose levels in vehicle-treated control and βJNK3-KO mice. Remarkably, improvement in glucose levels observed in Ex4 treated control mice was attenuated in the βJNK3-KO group (Figure 7A). The attenuated effect of Ex4 was observed after day 11 and persisted throughout the experiment, as demonstrated by a decrease in the AUC over 4 weeks of treatment (Figure 7B). The improvement in glucose homeostasis was accompanied by increases in random insulin levels in the Control + Ex4 group but not in the βJNK3-KO+Ex4 after 22 days of STZ administration (Figure 7C). The meal tolerance test was improved and insulin secretion was increased in controls treated with Ex4, and this effect was reduced in the βJNK3-KO +Ex4 group on day 16 (Figure 7, D–F). No difference in glucose tolerance was observed between vehicle-treated control and βJNK3-KO mice (Figure 7, D–F). Morphometric analysis demonstrated that the control group treated with Ex4 exhibited an increase in the number of islets per pancreatic section and β cell mass, and these responses were markedly decreased in βJNK3-KO+Ex4 mice (Figure 7, G–I). Control and βJNK3-KO treated with vehicle exhibited a comparable number of islets per pancreatic section and β cell mass (Figure 7, G–I).

### Effects of CTKs on stress-activated protein kinases (SAPKs) and c-Jun phosphorylation are reduced in βJNK3-KO islets.

To further assess the mechanisms of β cell mass preservation by Ex4 in the low-dose STZ model, we assessed the responses to proinflammatory cytokines. We first assessed the activation of SAPKs and downstream targets of JNKs by measuring different phosphorylation sites of c-JUN. Upon exposure to a cocktail of proinflammatory cytokines (Interleukin-1β, IFN-γ, TNF-α), control islets showed increased phosphorylation of SAPK (Thr183/Tyr185) at p54 and p46, and, consequently, phosphorylation of c-Jun at Ser63, Ser73, and Thr91 when exposed for 0.5 and 4 hours (Supplemental Figure 12, A and B). In contrast, islets from βJNK3-KO mice showed decreased phosphorylation of SAPK and c-JUN at Ser73 and Thr91 after cytokine treatment (Supplemental Figure 12, A and B).

### Protective effects of Ex4 against cytokine-induced apoptosis are mediated by JNK3.

**Figure 8:**
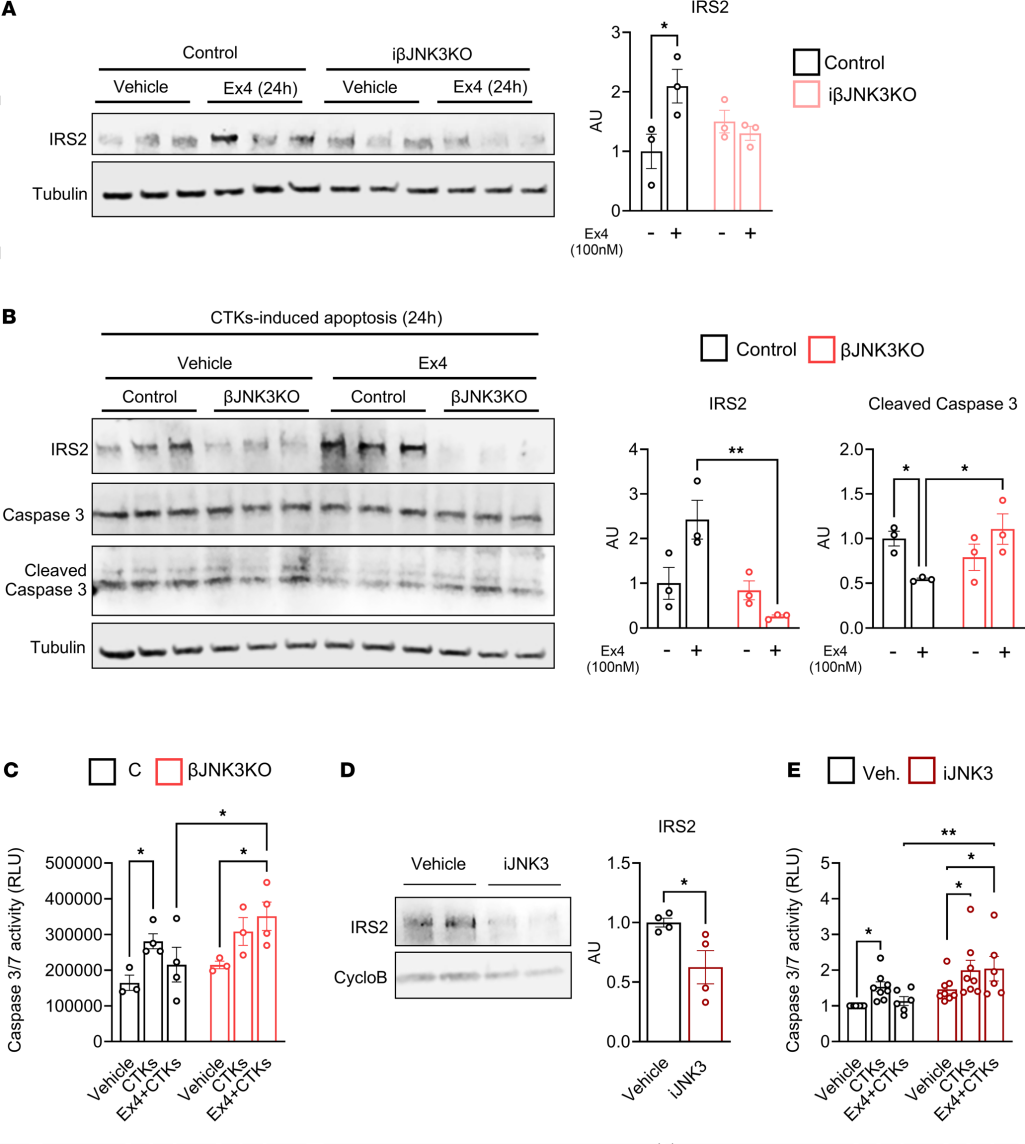
Protective effect of Ex4 against cytokine-induced apoptosis are mediated by JNK3. (**A**) IRS2 levels were measured after 24 hours of treatment with Exendin 4 in isolated islets from Control and βJNK3-KO. (**B**) IRS2 levels and CTK-induced apoptosis were measured by quantifying the cleaved caspase3 levels in presence or absence of Ex4 treatment. (**C**) Caspase 3/7 activity was evaluated in presence or absence of Ex4 and CTKs in islets from Control and βJNK3-KO. (**D**) IRS2 levels from donors no. 4–7 treated with vehicle or iJNK3 for 24 hours and (**E**) Caspase 3/7 activity from donors no. 6–7, 10–15 treated with vehicle or iJNK3 for 24 hours in presence or absence of Ex4 treatment. *P < 0.05, **P < 0.01, ***P < 0.001. The results are expressed as means ± SEM.

GLP1-R agonists trigger increases in cAMP-PKA-pCREB and stimulate the transcriptional regulation of key genes involved in β cell function and survival. Among these genes, Insulin Receptor Substrate 2 (IRS2) is a master regulator of survival in β cells (4, 15, 31). To evaluate if induction of the IRS2 protein by Ex4 is dependent on JNK3, we treated islets from control and iβJNK3-KO mice with Ex4. Protein levels of IRS2 increased in control islets and remained unchanged in iβJNK3-KO islets (Figure 8A). Given the role of JNK3 in activation of GLP-1R signaling and the known prosurvival effects of GLP-1R agonist against β cell apoptosis by regulation of IRS2 levels, we evaluated the role of JNK3 in GLP-1R agonist–mediated β cell survival by exposing control and βJNK3-KO islets to high glucose and cytokines for 24 hours in the presence or absence of Ex4. Ex4 treatment of control islets showed higher levels of IRS2 upon exposure to proinflammatory cytokines compared with vehicle-treated control islets and this was accompanied by lower levels of cleaved caspase 3 by immunoblotting and activity (Figure 8, B and C). In contrast, Ex4 failed to induce IRS2 levels in βJNK3-KO islets, and these islets lost the protection against apoptosis by Ex4, as assessed by Cleaved caspase 3 immunoblotting (Figure 8, B and C). Similar survival results were confirmed by measuring caspase 3/7 activity (Figure 8C). To test whether the survival signals are also modulated by JNK3 in human islets, we cultured human islets in the presence or absence of the JNK3 inhibitor. A decrease in IRS2 levels (Figure 8D) and an increase in caspase 3/7 activity induced by proinflammatory cytokines even under Ex4 (Figure 8E) were observed in islets treated with the JNK3 inhibitor, indicating that JNK3 mediates survival signals in human islets. Taken together, these studies indicate that the deletion of JNK3 impaired Ex4-mediated survival mechanisms in mouse and human islets.

## Discussion

In this study, we have identified the role of JNK3 in the incretin responses in β cells. Our findings show that βJNK3-KO mice exhibit impaired glucose homeostasis and insulin secretion following an oral glucose tolerance test, but not during an intraperitoneal glucose tolerance test, suggesting that JNK3 is required for incretin responses. We showed crosstalk between JNK3 and the cAMP/PKA/CREB signaling pathways by regulating *GLP1R* transcriptional levels and protein in mouse and human islets, supporting a role for JNK3 in potentiating insulin secretion in mouse and human islets. Finally, we demonstrated that the protective effect of Ex4 against apoptosis induced by proinflammatory cytokines and diabetes development in a preclinical model of diabetes was partially dependent on JNK3 in β cells, further underscoring the role of JNK3 in β cell function and survival.

Remarkably, database analysis of mouse single-cell transcriptome data (18) revealed dynamic expression patterns of JNK isoforms during development. Specifically, JNK3 exhibited low expression at the embryonic stage (E17) but showed a marked increase in expression from postnatal days 3–60. In contrast, JNK1 levels decreased over development, while JNK2 remained relatively stable. This pattern of JNK3 expression coincides with a period of increased β cell proliferation and maturation, suggesting a potential role for JNK3 in these processes and in optimizing responses to nutrients. Furthermore, analysis of single-cell transcriptomes from human donors revealed that JNK3 isoforms are specific to pancreatic cell types, with JNK3 being the most abundant isoform of JNKs in β cells. Also, JNK3 expression levels were found to be increased in donors with T1D and T2D and in a mouse model exposed to HFD. These findings indicate a direct involvement of JNK3 in β cell adaptation and responses to diabetes development. The data presented in this study highlight the involvement of JNK3 in incretin responses in both in vivo mouse models and human islets, suggesting the importance of JNK3 in human β cell biology and its potential as a therapeutic target in diabetes.

GLP-1R agonists are one of the most important medications in the treatment of T2D by enhancing insulin secretion and triggering survival pathways that preserve β cell mass (32). However, not all patients with T2D diabetes respond to these medications. Therefore, finding signaling pathways and mechanisms that can restore responses to GLP-1R agonists will have major implications for diabetes treatment. Our findings support a role for JNK3 in insulin secretory responses induced by GLP-1R agonists. JNK3 is required for proper incretin responses in β cells in a physiological context, as demonstrated in the oral glucose and meal tolerance tests, as well as in the progression of diabetes. Consistent with this, we showed that inhibition of JNK3 by pharmacological inhibitors or genetic JNK3 depletion decreases insulin secretion induced by GLP-1R agonists in vivo and in vitro by decreases in cAMP/PKA-CREB signaling. We discovered that the reduction of GLP-1 signaling was caused by decreased expression of GLP-1R and GLP-1R levels at the plasma membrane. These results are interesting because less is known about the regulation of GLP-1R transcription. Previous studies have shown that androgen receptor (Ar) binds to Ar motif elements and induced *Glp1r* transcription in mice (33). Analysis of the GLP-1R promoter region showed enrichment of putative binding sites for Jun transcription factors targeted by JNKs, suggesting that this could be a mechanism for reduction of *GLP1R* mRNA in β cells with inhibition of JNK3. Promoter region studies showed multiple putative c-Jun binding sites, and in vitro promoter-reporter assays demonstrated that JNK3 directly regulates the transcriptional activity of *GLP1R* gene. The possibility of JNK3 activation as a mechanism to potentiate GLP1 responses was then tested using transfection with gain-of-function plasmids. However, our gain-of-function experiments did not demonstrate a further increase in incretin responses. While this was disappointing, there are several limitations for the transfection experiments. Previous studies have shown that transient transfection procedures (including lipid-based or electroporation methods) may induce cellular stress responses, alter membrane properties (34), and transiently disrupt normal β cell physiology and signaling. These changes can affect the timing, amplitude, and biphasic pattern of insulin release. It remains possible that modulating JNK3 activity by small molecule activators could be leveraged to promote β cell resensitization to incretin signals and thereby enhance insulin secretion.

The potential of GLP-1R agonist acting on JNK3 to protect β cells in diabetogenic conditions raises intriguing questions. Kinase activity profiles of human pancreatic β cells downstream of GLP-1R have shown that biased agonists exendin-asp3 differentially modulate JNK3 activity (35). Interestingly, exendin-asp3 is associated with preferential β arrestin recruitment, suggesting that this biased agonist can activate JNK3 via β arrestin. This is consistent with previous data identifying JNK3 as a binding partner of β arrestin 2 using a yeast 2-hybrid screen and in vitro studies (36–38). β arrestin 2 and arrestin 3 are known to be involved in MAPK signaling, and deletion of β arrestin 2 in β cells has been shown to impair insulin secretion, primarily due to impaired CAMKII function in β cells (36). This is consistent with the concept that β arrestin 2 acts as a scaffold protein that brings the spatial distribution and activity of JNK3 under the control of G protein–coupled receptors (38). β arrestin 2 has been implicated in incretin sensitivity in β cells by promoting posttranslational modification of GLP-1R trafficking, leading to prolonged GLP-1R signaling (39). While β arrestin 2 can bind to JNK3 in the cytoplasm, its role in regulating JNK3 activity remains unclear. On the other hand, arrestin 3 exhibits a 15-fold higher affinity for the inactive form of JNK3 compared with the active form, and its release upon MKK7 phosphorylation is crucial for signal amplification (40). Taken together, the published evidence and our studies lead us to speculate that GLP-1 activates JNK3 activity via β arrestins and regulates GLP-1R expression, and, consequently, JNK3 inhibition indirectly downregulates GLP-1 signaling. Our results are also consistent with a model in which deletion of JNK3 can indirectly regulate the cAMP/PKA axis downstream of GLP-1 signaling by modulation of GLP-1R expression.

One unifying characteristic of patients with both major forms of diabetes is the loss of functional β cell mass (1). GLP-1R agonists have been explored as therapeutic strategies to increase and/or preserve β cell mass in diabetes, aiming to maintain functional β cells and induce their survival and regeneration (41). These pharmacological agents protect β cells against apoptosis in vitro and in in vivo, in models of loss of β cells induced by multiple low-dose STZ injections (42, 43). In contrast, the finding that GLP-1R deletion in β cells (42) or the use of Ex9, an antagonist of the GLP-1R (44), leads to enhanced susceptibility to STZ-induced apoptosis, demonstrates that GLP-1 receptor signaling is an important physiological determinant of β cell survival in a preclinical model of diabetes. Similarly, our studies show that JNK3 depletion in islets reduced the beneficial effects of Ex4 on glucose homeostasis and glucose tolerance in the low-dose STZ model, and this was explained in part by impairment of β cell survival in Ex4-treated βJNK3-KO mice (Figure 7, G–I). The difference in mixed meal tolerance between normoglycemic βJNK3-KO and STZ-treated βJNK3-KO mice likely reflects the marked β cell loss in the STZ model, which can mask the glucose intolerance phenotype evident in the inducible model under basal conditions. Mechanistically, this was caused by reduced GLP-1R expression and cAMP/PKA/CREB axis signaling, ultimately resulting in decrease in IRS2 levels (Figure 8). Taken together, these studies support a function for JNK3 in mediating the beneficial effects of GLP-1 analogs in a model of β cell destruction and suggest that this pathway could be used to sensitize or amplify β cell responses to incretins.

Recent case reports show that the GLP-1R agonist semaglutide has beneficial effects in newly diagnosed patients with T1D. The in vivo survival effects observed in the STZ model were further explored in vitro using a cocktail of CTKs that mimic the T1D environment. CTK treatment induction of c-Jun phosphorylation was reduced in βJNK3-KO mice, and the protective effect of Ex4 in alleviating apoptosis induced by proinflammatory cytokines was lost after the deletion of JNK3 in mouse islets (Figure 8). Whether this effect is fully explained by the decrease in GLP-1R expression or involves other associated signaling pathways remains an open question. Importantly, the survival effects induced by Ex4 were reduced by JNK3 inhibition in mouse and human islets (Figure 8, B–E). The decreased survival by inhibition of JNK3 was associated with reduced levels of IRS2 in mouse and human islets (Figure 8). This is consistent with previous data showing that JNK3 silencing in insulinoma cells decreases IRS2 expression (45) and sensitizes cells to cytokine-induced apoptosis even after treatment with Ex4 (4, 46). In summary, our results position JNK3 as a key component in regulation of β cell survival in mouse and human islets and identify this kinase as a major step in survival responses to GLP-1R analogs by controlling expression of IRS2. In addition, the increased JNK3 expression levels in islets from in donors with T1D and T2D underscore the importance of JNK3 in human β cells and its potential as a therapeutic target in diabetes.

In conclusion, the possibility of activating JNK3 to sensitize β cells to the effects of GLP-1R analogs on insulin secretion and β cell survival in diabetogenic conditions raises intriguing opportunities of this kinase as a potential therapeutic target. Future studies can be designed to test the combination of GLP-1R analog–based therapy in combination with JNK3 activators for the treatment of T1D and T2D.

## Methods

### Sex as a biological variable.

Both male and female mice were included in this study. Metabolic and molecular outcomes are presented separately by sex, and no sex-specific differences were observed in the measured parameters. Because the pathways examined are fundamental to β cell biology and glucose homeostasis, the findings are expected to be relevant to both sexes. Sex was not used as an experimental variable in statistical analyses.

### Animals.

*JNK3* mice harboring a floxed *MAPK10* (JNK3) allele (also named *JNK3^fl/fl^*) were described previously (47). Deletion of *JNK3* in the pancreatic β cells was achieved by intercrossing *JNK3^fl/fl^* mice with the Ins1-cre mouse (22). Male offspring positive for the *INS-Cre* transgene carrying 2 floxed *JNK3* alleles (*Ins-Cre*;*JNK^fl/fl^*) were analyzed and, for simplicity, are referred to as βJNK3-KO mice. To exclude any effect of the *Ins-Cre* or *Mip-Cre^Ertm^* transgene, we performed glucose tolerance test in animals harboring the floxed gene, *Mip-Cre^Ertm^*, and no differences were observed (48). Therefore, male littermates negative for the *Ins-Cre* transgene (*JNK3^fl/fl^*) or Ins-Cre alone were used as controls for all experiments. Inducible deletion of *JNK3* was achieved by crossing *JNK^fl/fl^* mice with *Tg(MIP1-Cre/ERT)^1Lph^* mice (*Mip-Cre^Ertm^*), which express Cre recombinase under the control of a mouse insulin (*Ins1*) promoter in a tamoxifen-inducible manner (*i*βJNK3-KO mice) (48). Mice received 3 tamoxifen (Tamx) injections (2 mg/kg, every other day). All procedures were performed in accordance with the University Committee on the Use and Care of Animals at the University of Miami.

### Metabolic studies.

Glucose was measured in whole blood using ACCU-CHEK II glucometer (Roche). Plasma insulin levels were measured using a rat insulin ELISA kit (ALPCO Immunoassays). Glucose tolerance was assessed by measuring blood glucose levels following administration of 2 g/kg glucose by either i.p. injection or oral gavage in mice fasted for 5 hours. Incretin response was determined by coadministration of 1 nmol/kg of Exendin 4 (Ex4) (Sigma) with 2 g/kg glucose intraperitoneally followed by measurement of blood glucose and plasma insulin. Meal tolerance test was assessed by oral gavage of 10 ml/kg body weight of Ensure (liquid, Abbott Laboratories).

### Multiple low-doses of STZ.

Diabetes was induced by five i.p. low-dose injections of STZ (Sigma, 40 mg/kg) (30). Treatment with GLP-1R agonist Exendin 4 (Ex4) was intraperitoneally administrated at 1 nmol/kg 2 days before STZ treatment by 4 weeks. Blood glucose levels were determined 3 times per week in blood obtained from the tail vein using ACCU-CHEK II glucometer (Roche).

### MIN6 cells.

MIN6 cells were obtained from the laboratory of Armando Mendes at the Diabetes Research Institute, University of Miami, and were maintained under standard culture conditions

### Mouse and human islets.

Mouse islet isolation was performed as previously described (24). Briefly, the pancreas was inflated with 1 mg/mL collagenase P (Roche) injected into the common duct. Islets were handpicked and incubated in a 37°C humidified chamber overnight in RPMI containing 10% FBS, 1% penicillin/streptomycin, and 5.5 mM glucose prior to performing subsequent experiments. Human islets were derived from seven donors; see Human Islet Checklist in Supplemental Table 1 adapted from (49) from ProdoLab and nPOD. Human islets were treated with iJNK3 for 24 hours prior to performing subsequent experiments.

For static insulin secretion, isolated islets were incubated in Kreb’s buffer (114 mM NaCL, 4.7 mM KCl, 1.2 mM KH2PO4, 1.16 mM MgSO4, 20 mM HEPES, 2.5 mM CaCl2, 25.5 mM NaHCO3, and 0.2% BSA, pH 7.2) containing 2 mM glucose for 2 hours. Groups of 20 islets in were incubated in Krebs-Ringer medium containing 2 mM or 16.7 mM glucose for 0.5 hours with or without Ex4 (100 nM) was added when indicated. Secreted insulin was then measured in the media using the Ultrasensitive Insulin ELISA kit (ALPCO Immunoassays) and normalized to total insulin content.

### Proinflammatory cytokine–induced apoptosis in vitro.

Cytokine-induced apoptosis was performed by treating mouse islets with human interleukin-1β (50 U/mL), recombinant rat IFN-γ (1,000U/mL), and recombinant rat tumor necrosis factor-α (1,000 U/mL) (Peprotech) in the presence of high glucose concentration using 16.7 mM for mouse islets (13). Caspase-Glo 3/7 Assay (Promega) was used to measure the Caspase3/7 activity 24 hours after treatments. Background signal (media-only wells) was subtracted from all values. Fold change was calculated based on the control treated with vehicle.

### Luxendin labeling in live islets.

Isolated islets were incubated with 100 nM LUXendin551 (28) for the indicated times at 37°C in culture medium. Following incubation, islets were washed 3 times with KRB and imaged using a Leica TCS SP8 confocal microscope. Excitation/emission settings were as follows: LUXendin551, λ = 561 nm / 569–667 nm.

### Plasmid-mediated JNK overactivation (gain-of-function).

Plasmid expression vectors encoding fusion proteins with an NH_2_-terminal Flag epitope tag and JNK isoforms were generated as previously described (50). The MKK7–JNK fusion proteins consisted of residues 1–443 of MKK7 fused to JNK1α1 (1–383), JNK2α2 (1–423), or JNK3α2 (1–463). Point mutations were introduced to create phosphorylation-negative JNK1 (Thr180–Pro–Tyr182 replaced with Ala–Pro–Phe, APF), which prevents phosphorylation when fused to constitutively active MKK7 (MKK7–JNK1APF).

### Luciferase reporter assay.

The approximately 1500 bp promoter region of the human *GLP1R* gene was synthesized, sequence-verified, and cloned into the pMCS-Red Firefly Luc Vector (ThermoFisher). HEK293T cells were seeded into 24-well plates and grown to 70%–80% confluence before transfection with pCDNA3.1-MKK7–JNK1 or pCDNA3.1-MKK7–JNK3 (for JNK overactivation), pCDNA3.1–GLP1R, and pRL-TK (Renilla luciferase control) using XTreme transfection reagent (Roche). After 24 hours, cells were incubated for 16 hours in DMEM (4.5 g/L glucose, 10% FBS, 1% antibiotics) containing either vehicle (DMSO) or iJNK3 (5 μM) at 37°C with 5% CO_2_. Cells were then washed with ice-cold PBS and lysed for luciferase assays. Firefly and Renilla luciferase activities were measured sequentially using the Luciferase Reporter Assay System (Promega) according to the manufacturer’s instructions. Firefly luciferase activity was normalized to Renilla luciferase to account for transfection efficiency, and results were expressed as relative luminescence units (RLU).

### RT-PCR.

Total RNA was isolated from mouse and human islets using the RNeasy isolation kit (Qiagen). Gene expression was performed by q-RT-PCR using Power SYBR Green PCR Mix (Applied Biosystems) using Quant Studio 3 Real-Time PCR systems (Applied Biosystems) with a standard protocol including a melting curve. The PCR reactions were performed following the kit instructions. Primers from IDT Technologies (Supplemental Table 3) were used. Relative abundance for each transcript was calculated by a standard curve of cycle thresholds and normalized to housekeeping gene.

### Western blot.

Twenty to 30 micrograms of protein lysate from islets in lysis buffer (10 mM Tris-HCl, 1% SDS, anti-proteases and anti-phosphatases) (Roche) were resolved in polyacrylamide gel and transferred to a polyvinylidene fluoride membrane (Bio-Rad). The membrane was then blotted with the antibodies described in Supplemental Table 2 and visualized using the Li-Cor system (Li-Cor). Densitometry was determined by measuring pixel intensity using NIH Image J software and normalized to tubulin in the same membrane. Goat anti-mouse and anti-rabbit secondary antibodies were from LI-COR Biosciences.

### Immunofluorescence and morphometry.

Pancreata were fixed overnight in formalin (4% formaldehyde) and embedded in paraffin as previously described (13). Antigen retrieval was achieved by boiling in Citrate Buffer (10 mM NaCitrate, pH 6.0) for 4–12 minutes. Nonspecific binding was blocked by incubating with 5% goat serum for 30 minutes and then sections were incubated at 4°C overnight with antiinsulin primary antibody (Dako), followed by incubation with fluorophore-conjugated secondary antibodies (Jackson Immunoresearch). The antibodies are described in Supplemental Table 1. Coverslips were mounted on slides using DAPI-containing mounting media (Vector Laboratories). Assessment of β cell mass was performed by measuring insulin and acinar areas from 4 insulin-stained sections separated by 200 μM, using NIH Image J Software (v1/49d available free at http://rsb.info.nih.gov/ij/index.html). The ratios of the hormone-stained area to the total pancreatic section area for each mouse were averaged and multiplied by the pancreatic weight.

### Statistics.

Data are presented as mean ± SEM. The statistical significance of differences between the various conditions was determined by nonparametric U test (Mann-Whitney). Two-way ANOVA was used to detect differences between groups over experimental condition followed by a Tukey’s (or Šídák’s) multiple comparison test using Prism version 10 (GraphPad Software). Results were considered statistically significant when the *P* value was less than 0.05.

### Study approval.

All procedures involving mice were conducted in accordance with the ARRIVE guidelines and approved by the Institutional Animal Care and Use Committee (IACUC) of the University of Miami (Protocol #IPROTO202100000125). The use of Human islets was reviewed and approved by the University of Miami Institutional Review Board, and all methods were carried out in accordance with institutional guidelines and the U.S. Department of Health and Human Services regulations (45 CFR 46). No identifiable images or information from human subjects are included.

### Data availability.

All data supporting the findings of this study are provided in the article, the supplemental materials, and the Supporting Data Values file. Additional datasets generated and/or analyzed during the current study are available from the corresponding authors upon reasonable request. Previously published single-cell RNA-seq datasets used in this study are available at ArrayExpress (E-MTAB-5061, E-MTAB-5060) and through the HPAP PANC-DB portal.

## Author contributions

RAL and EBM designed research. RAL, MGM, VPG, JB, AFN, JA, and MBR performed research. RAL, MGM, VPG, JB, AFN, JA, MSH, RJD, MBR, GL, and EBM analyzed data. RAL, GL, MSH, RJD, and EBM reviewed the paper.

## Funding support

This work is the result of NIH funding, in whole or in part, and is subject to the NIH Public Access Policy. Through acceptance of this federal funding, the NIH has been given a right to make the work publicly available in PubMed Central.

- NIH, United States: R01-DK073716 (EBM), R01-DK132103 (EBM), R01-DK133183 (EBM), R01-DK138471 (EBM), and R56-DK138005 (MSH).
- The Medical Research Service of the Department of Veterans Affairs (EBM).
- The Division of Endocrinology, Diabetes and Metabolism at the University of Miami Miller School of Medicine.

## Supplementary Material

Supplemental data

Unedited blot and gel images

Supporting data values

## Version 1 01/02/2026

Electronic publication

## References

[1] Atkinson MA (2015). Current concepts on the pathogenesis of type 1 diabetes--considerations for attempts to prevent and reverse the disease. Diabetes Care.

[2] Abdelli S (2004). Intracellular stress signaling pathways activated during human islet preparation and following acute cytokine exposure. Diabetes.

[3] Hotamisligil GS, Davis RJ (2016). Cell Signaling and Stress Responses. Cold Spring Harb Perspect Biol.

[4] Abdelli S (2009). JNK3 is abundant in insulin-secreting cells and protects against cytokine-induced apoptosis. Diabetologia.

[5] Ammendrup A (2000). The c-Jun amino-terminal kinase pathway is preferentially activated by interleukin-1 and controls apoptosis in differentiating pancreatic beta cells. Diabetes.

[6] Varona-Santos JL (2008). c-Jun N-terminal kinase 1 is deleterious to the function and survival of murine pancreatic islets. Diabetologia.

[7] Bonny C (2001). Cell-permeable peptide inhibitors of JNK: novel blockers of beta-cell death. Diabetes.

[8] Bennett BL (2003). JNK: a new therapeutic target for diabetes. Curr Opin Pharmacol.

[9] Lee YH (2003). c-Jun N-terminal kinase (JNK) mediates feedback inhibition of the insulin signaling cascade. J Biol Chem.

[10] Barshes NR (2005). Inflammation-mediated dysfunction and apoptosis in pancreatic islet transplantation: implications for intrahepatic grafts. J Leukoc Biol.

[11] Lanuza-Masdeu J (2013). In vivo JNK activation in pancreatic β cells leads to glucose intolerance caused by insulin resistance in pancreas. Diabetes.

[12] Kim WH (2005). Synergistic activation of JNK/SAPK induced by TNF-alpha and IFN-gamma: apoptosis of pancreatic beta cells via the p53 and ROS pathway. Cell Signal.

[13] Louzada RA (2023). GHRH agonist MR-409 protects β cells from streptozotocin-induced diabetes. Proc Natl Acad Sci U S A.

[14] Hennige AM (2003). Upregulation of insulin receptor substrate-2 in pancreatic beta cells prevents diabetes. J Clin Invest.

[15] Jhala US (2003). cAMP promotes pancreatic beta-cell survival via CREB-mediated induction of IRS2. Genes Dev.

[16] Abdelli S, Bonny C (2012). JNK3 maintains expression of the insulin receptor substrate 2 (IRS2) in insulin-secreting cells: functional consequences for insulin signaling. PLoS One.

[17] Tenenbaum M (2021). The Map3k12 (Dlk)/JNK3 signaling pathway is required for pancreatic beta-cell proliferation during postnatal development. Cell Mol Life Sci.

[18] Qiu WL (2017). Deciphering pancreatic islet β cell and α cell maturation pathways and characteristic features at the single-cell level. Cell Metab.

[19] Segerstolpe A (2016). Single-cell transcriptome profiling of human pancreatic islets in health and type 2 diabetes. Cell Metab.

[20] Elgamal RM (2023). An integrated map of cell type-specific gene expression in pancreatic islets. Diabetes.

[21] Blandino-Rosano M (2023). Raptor levels are critical for β cell adaptation to a high-fat diet in male mice. Mol Metab.

[22] Thorens B (2015). Ins1(Cre) knock-in mice for beta cell-specific gene recombination. Diabetologia.

[23] Herrera PL (1998). Two transgenic approaches to define the cell lineages in endocrine pancreas development. Mol Cell Endocrinol.

[24] Blandino-Rosano M (2017). Loss of mTORC1 signalling impairs β cell homeostasis and insulin processing. Nat Commun.

[25] El K (2021). GIP mediates the incretin effect and glucose tolerance by dual actions on α cells and β cells. Sci Adv.

[26] Kamenecka T (2009). Structure-activity relationships and X-ray structures describing the selectivity of aminopyrazole inhibitors for c-Jun N-terminal kinase 3 (JNK3) over p38. J Biol Chem.

[27] Van de Velde S (2019). CREB promotes beta cell gene expression by targeting its coactivators to tissue-specific enhancers. Mol Cell Biol.

[28] Ast J (2022). Expanded LUXendin color palette for GLP1R detection and visualization in vitro and in vivo. JACS Au.

[29] Zeke A (2016). JNK signaling: regulation and functions based on complex protein-protein partnerships. Microbiol Mol Biol Rev.

[30] Ito M (2001). Characterization of low dose streptozotocin-induced progressive diabetes in mice. Environ Toxicol Pharmacol.

[31] Withers DJ (1998). Disruption of IRS-2 causes type 2 diabetes in mice. Nature.

[32] Drucker DJ (2018). Mechanisms of action and therapeutic application of glucagon-like peptide-1. Cell Metab.

[33] Zhu L (2019). Glucagon-like peptide-1 receptor expression and its functions are regulated by androgen. Biomed Pharmacother.

[34] Li Z (2019). Lipofectamine 2000/siRNA complexes cause endoplasmic reticulum unfolded protein response in human endothelial cells. J Cell Physiol.

[35] Xiao J (2023). Control of human pancreatic beta cell kinome by glucagon-like peptide-1 receptor biased agonism. Diabetes Obes Metab.

[36] Perry-Hauser NA (2022). Short arrestin-3-derived peptides activate JNK3 in cells. Int J Mol Sci.

[37] Zhan X (2016). Peptide mini-scaffold facilitates JNK3 activation in cells. Sci Rep.

[38] McDonald PH (2000). Beta-arrestin 2: a receptor-regulated MAPK scaffold for the activation of JNK3. Science.

[39] Jones B (2018). Targeting GLP-1 receptor trafficking to improve agonist efficacy. Nat Commun.

[40] Perry NA (2019). Arrestin-3 scaffolding of the JNK3 cascade suggests a mechanism for signal amplification. Proc Natl Acad Sci U S A.

[41] Aguayo-Mazzucato C, Bonner-Weir S (2018). Pancreatic β cell regeneration as a possible therapy for diabetes. Cell Metab.

[42] Li Y (2003). Glucagon-like peptide-1 receptor signaling modulates beta cell apoptosis. J Biol Chem.

[43] Maida A (2009). Differential importance of glucose-dependent insulinotropic polypeptide vs glucagon-like peptide 1 receptor signaling for beta cell survival in mice. Gastroenterology.

[44] De Leon DD (2003). Role of endogenous glucagon-like peptide-1 in islet regeneration after partial pancreatectomy. Diabetes.

[45] Prause M (2016). JNK1 deficient insulin-producing cells are protected against interleukin-1β induced apoptosis associated with abrogated Myc expression. J Diabetes Res.

[46] Ezanno H (2014). JNK3 is required for the cytoprotective effect of exendin 4. J Diabetes Res.

[47] Vernia S (2016). Excitatory transmission onto AgRP neurons is regulated by cJun NH2-terminal kinase 3 in response to metabolic stress. Elife.

[48] Blandino-Rosano M (2022). Novel roles of mTORC2 in regulation of insulin secretion by actin filament remodeling. Am J Physiol Endocrinol Metab.

[49] Hart NJ, Powers AC (2019). Use of human islets to understand islet biology and diabetes: progress, challenges and suggestions. Diabetologia.

[50] Lei K (2002). The Bax subfamily of Bcl2-related proteins is essential for apoptotic signal transduction by c-Jun NH(2)-terminal kinase. Mol Cell Biol.

