# Supplementary material for: JNK3 regulates β cell responses to incretins in human islets and mouse models
Source: J Clin Invest. 2026 Jan 2;136(1):e185707. doi: 10.1172/JCI185707 (PMC12721909; doi:10.1172/JCI185707)
Supplement: Supplemental data [file jci-136-185707-s188.pdf]

Supplemental Information

JNK3 regulates in β-cell responses to incretins in human islets and mouse models

Ruy A. Louzada[1 §], Marel Gonzalez Medina[1], Valentina Pita Grisanti[1], Jessica Bouviere[2], Amanda F. Neves[1], Joana Almaça[1], Myoung S. Han[3], Roger Davis[3], Gil Leibowitz[4], Manuel Blandino-Rosano[1], and Ernesto Bernal-Mizrachi[1,5 §]

[1] Division of Endocrinology, Diabetes, and Metabolism, Department of Medicine, University of Miami Miller School of Medicine, Miami, FL

[2] Department of Biochemistry and Molecular Biology, University of Miami Miller School of Medicine, Miami, FL

[3] Program in Molecular Medicine, University of Massachusetts Chan Medical School, Worcester, MA, USA

[4] Diabetes Unit and Endocrine Service, Hadassah-Hebrew University Medical Center, Jerusalem, Israel.

[5] Veterans Affairs Medical Center, Miami, Florida, USA

Figure S1

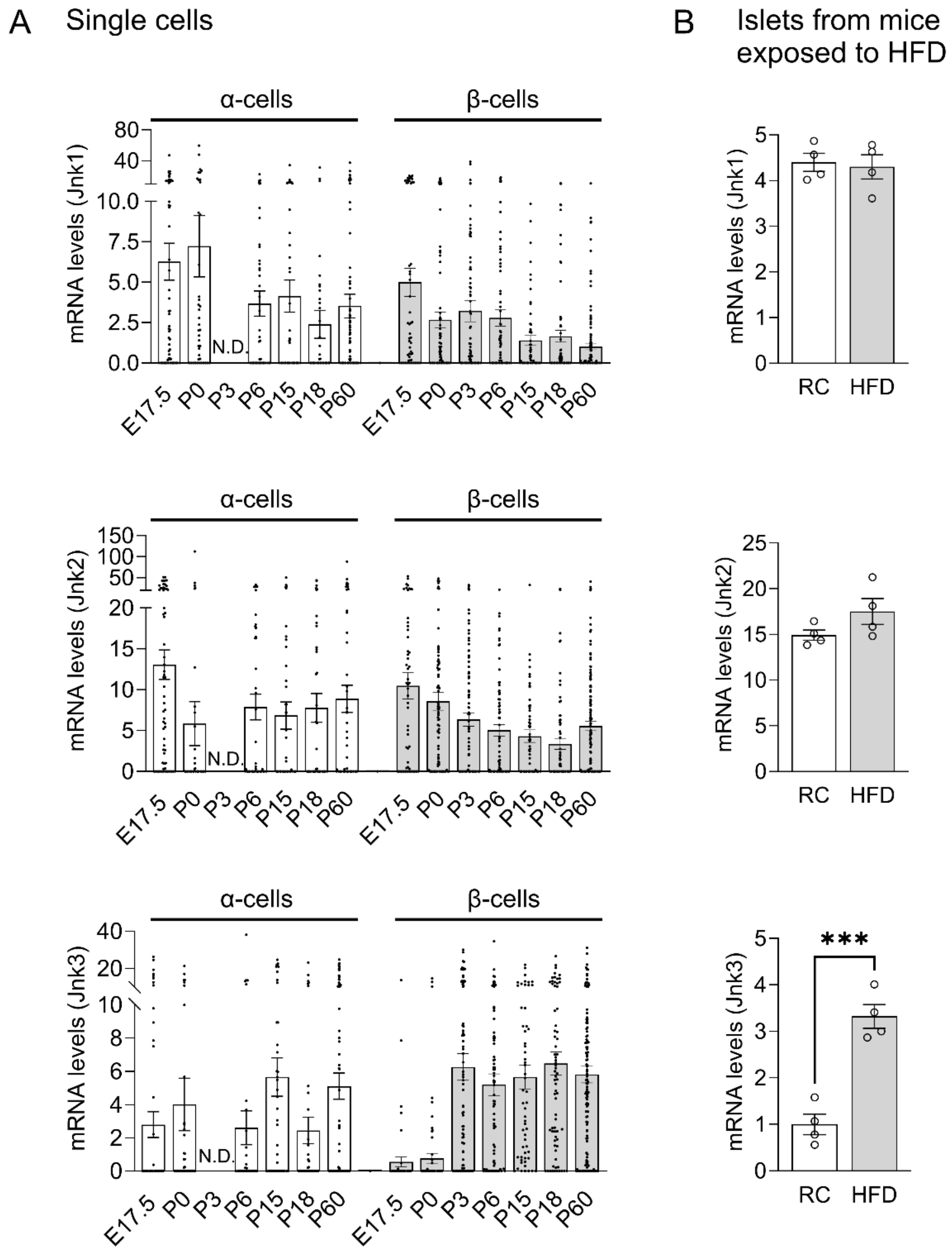

Supplemental Figure 1 (S1). JNK3 mRNA is the most abundant JNK isoform in β-cells (18, 21).

Figure S2

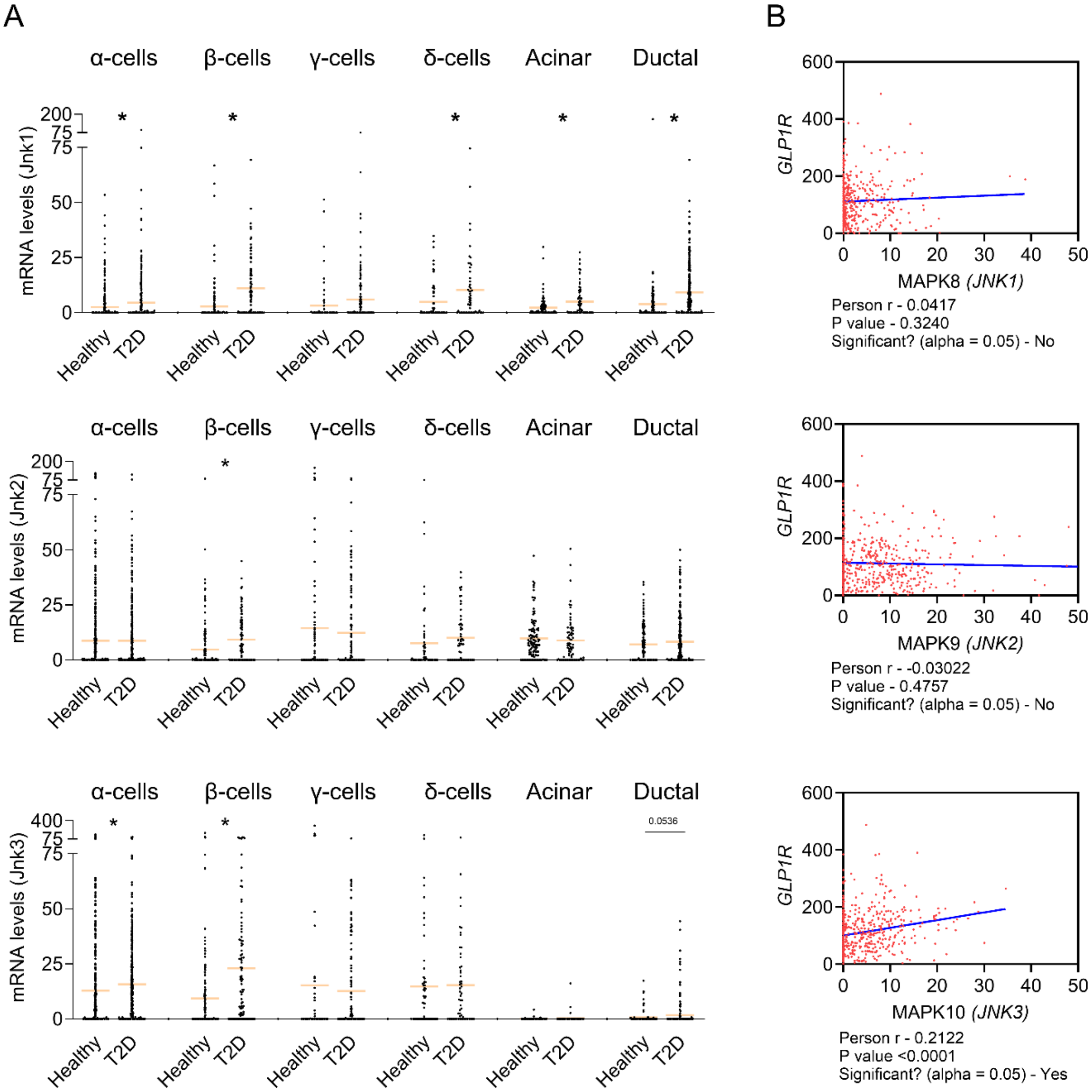

Supplemental Figure 2 (S2). JNK mRNA levels are increased in human diabetes (19). A) JNK expression in α, β, δ, γ, ductal, and acinar cells. B) Correlation between JNK isoforms and GLP1R expression. Single-cell RNA-seq data were obtained from publicly available datasets (ArrayExpress accessions E-MTAB-5061 and E-MTAB-5060)

Figure S3

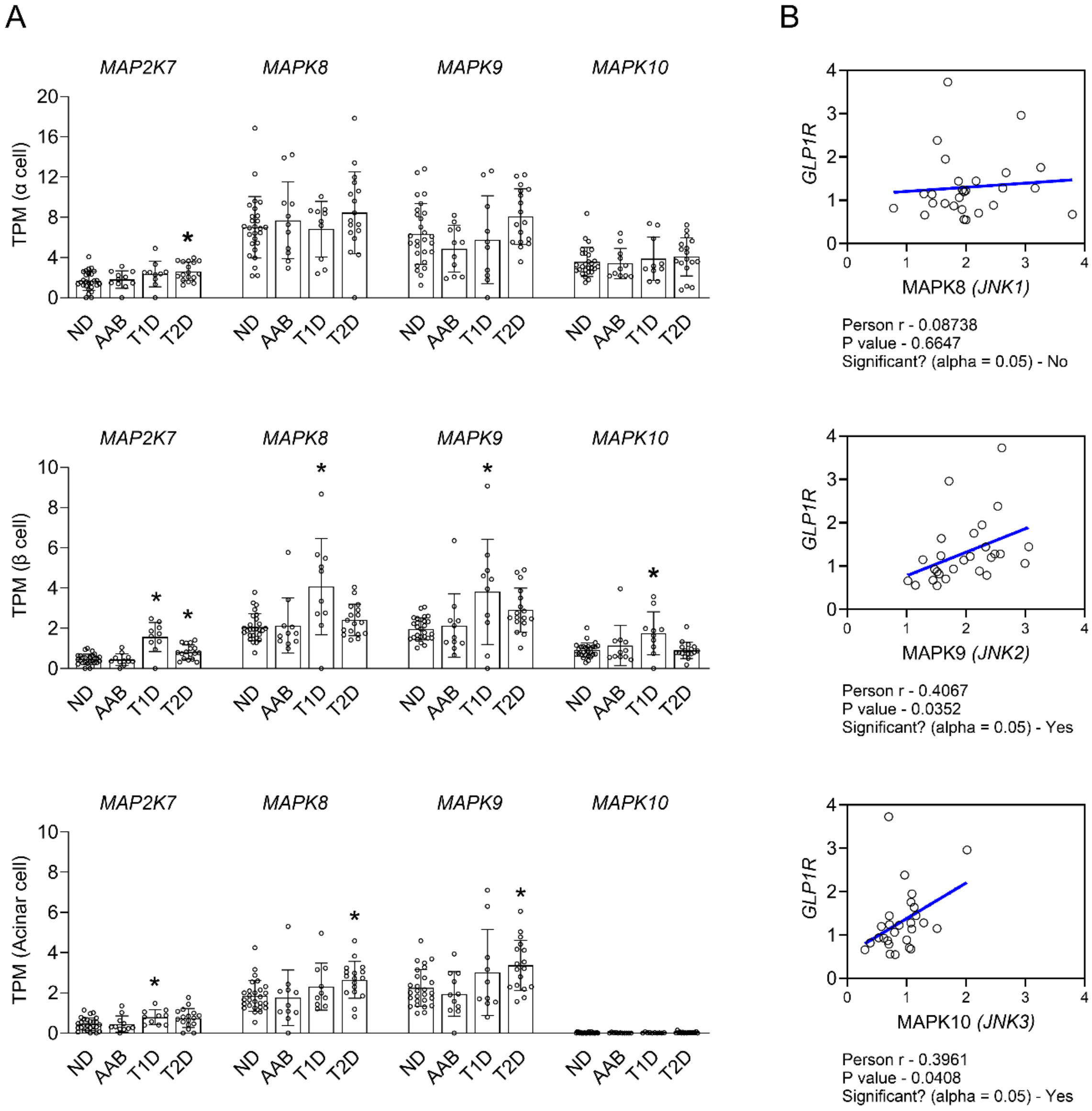

Supplemental Figure 3 (S3). JNKs mRNA are induced in human diabetes (20). A) JNK expression in α, β, and acinar cells. B) Correlation between JNK isoforms and GLP1R expression. Single-cell RNA-seq data were obtained from the publicly available HPAP human islet reference map (HPAP PANC-DB).

Figure S4

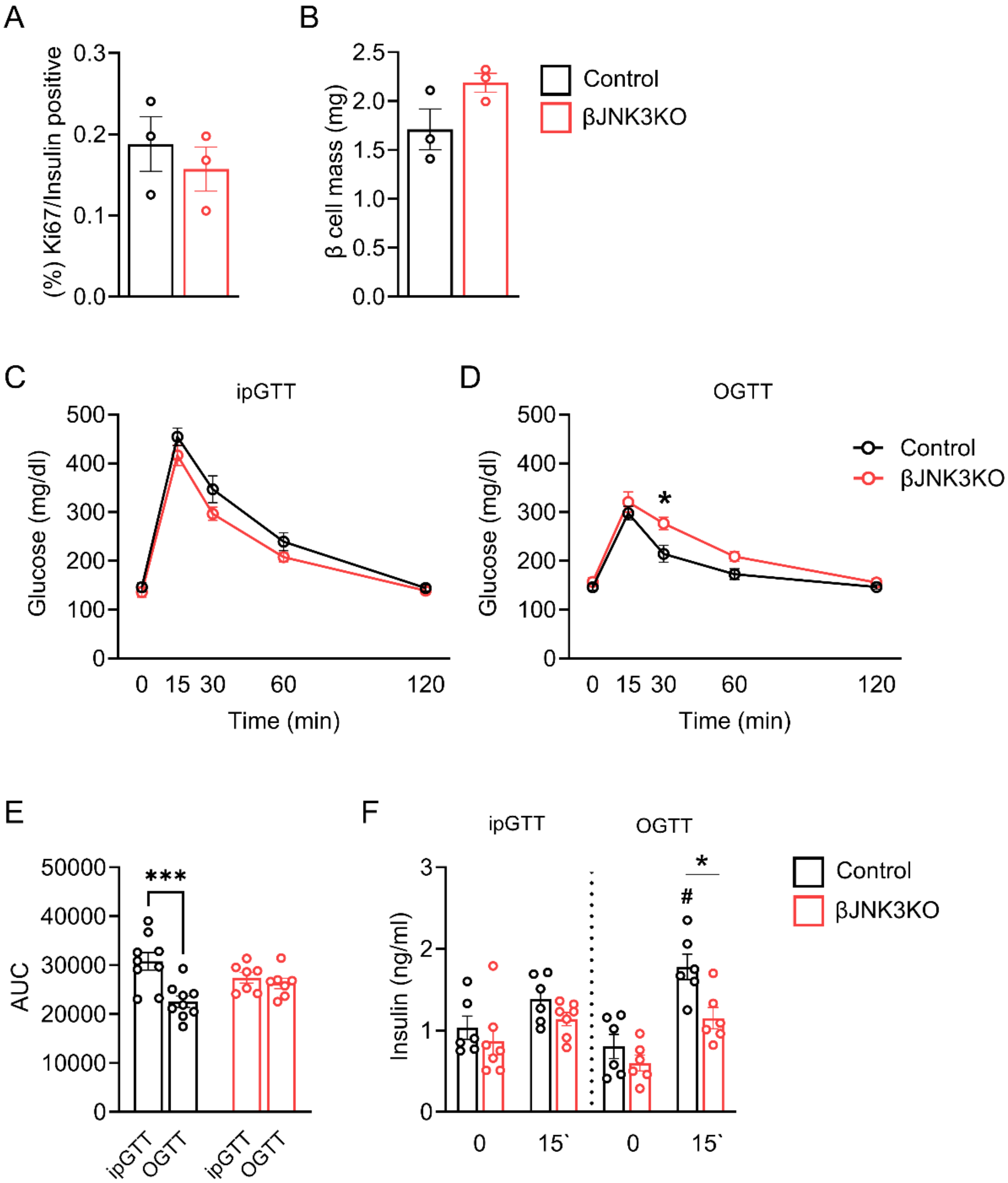

Supplemental Figure 4 (S4). Deletion of JNK3 in β-cells results in glucose intolerance and defective insulin secretion in female mice. A) Intraperitoneal glucose tolerance test (ipGTT). B) Oral glucose tolerance test (OGTT). C) AUC calculations for glucose tolerance tests. D) Insulin levels at baseline and 15 minutes after glucose challenge. E) Ki67/insulin co-staining in pancreatic sections from 6-month-old males. F) β-cell mass quantification. Data are expressed as means ± EM. Statistical significance was determined by Two-way ANOVA. *P < 0.05, ***P < 0.001, ***P < 0.0001 between groups; #P < 0.05 within the same group.

Figure S5

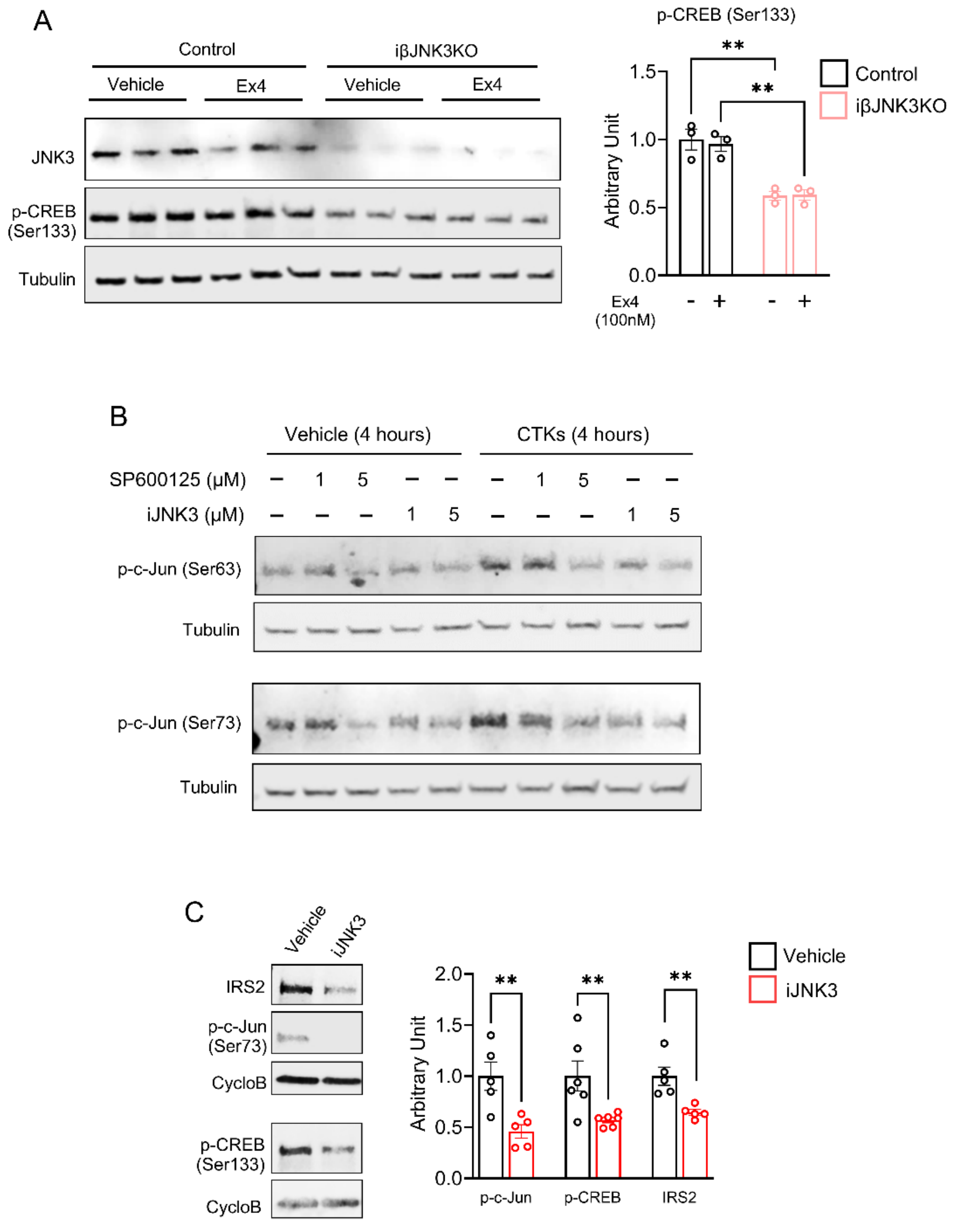

Supplemental Figure 5 (S5). A) JNK3 levels and phosphorylation of CREB in the iβJNK3KO model. A) JNK3 and phosphorylation of CREB levels in isolated islets from Control and iβJNK3KO at 6-month-old male mice. B) Pharmacological inhibition of JNK3 (iJNK3) in mouse islets and MIN6 cells. A) Phosphorylation of c-Jun at Serine 63 and Serine 73 were assessed by immunoblotting of isolated islets from 4-6 months old Control male mice treated with proinflammatory cytokines for 4 hours in presence or absence of iJNK3 and SP600125. B) IRS2, phosphorylation of CREB and phosphorylation of c-Jun at Serine 73 levels in MIN6 treated with JNK3 inhibitor (iJNK3). The α-Tubulin loading control shown here is reused from Figure 8A, as both blots were run on the same gel and represent the same experiment.

Figure S6

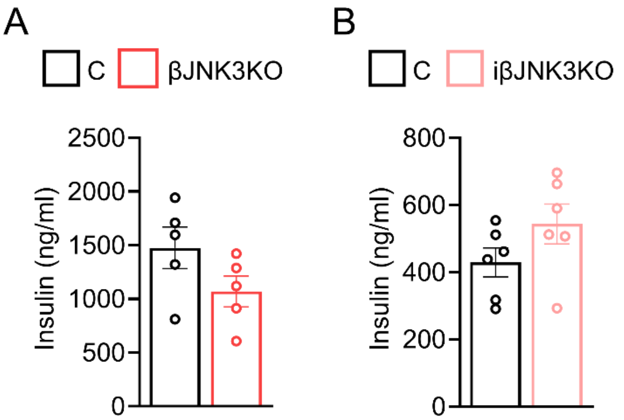

C

Total Content of GSIS		
Human Insulin (pmol)		
Donor	Vehicle	iJNK3
#4	21451	25979
#5	6242	3687
#6	9188	11473
#8	34758	31408
#9	35535	32158
#10	31286	36151
#15	12925	11698

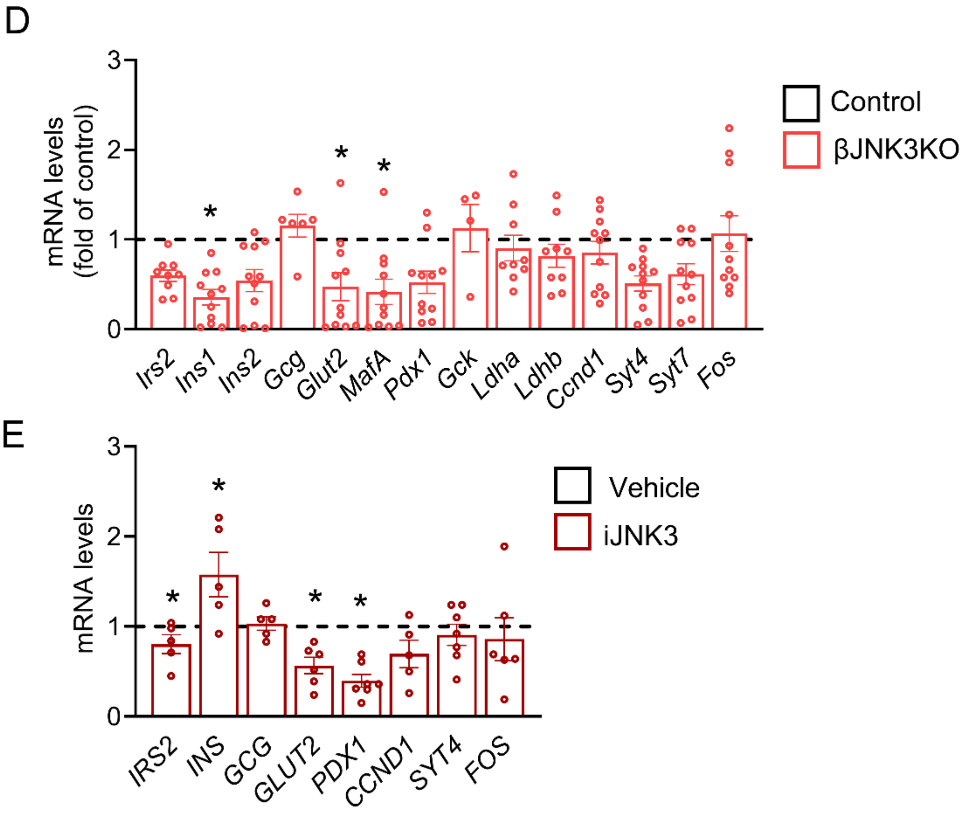

Supplemental Figure 6 (S6). Total insulin content from GSIS assays and RT-PCR: A) βJNK3KO, B) iβJNK3KO, C) human islets (donors #4-6, 8-10, 15). D) RT-PCR for β-cell identity and CREB-responsive genes in control and βJNK3KO islets. E) RT-PCR for β-cell identity and CREB-responsive genes in human islets treated with iJNK3 (Donors #1-5, 8).

Figure S7

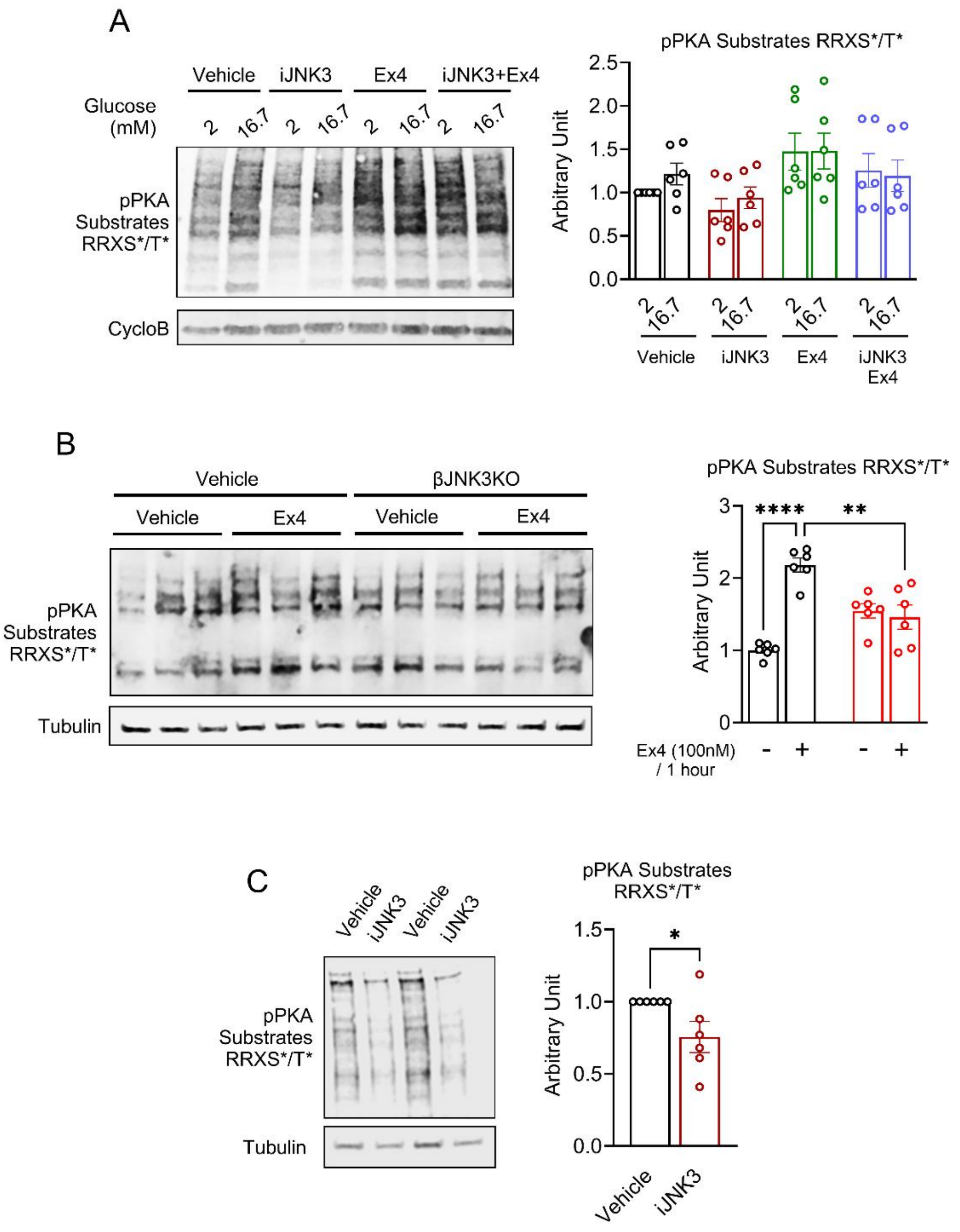

Supplemental Figure 7 (S7). PKA activity following Exendin 4 treatment. A) MIN6 cells ± iJNK3. The Cyclophilin B loading control shown here is the same blot presented in Figure 4A, as both panels were derived from the same experiment and gel. B) Islets from 4–6-month-old control and βJNK3KO male and female mice. C) Human islets treated with vehicle or iJNK3 (donors #1, 3-7).

Figure S8

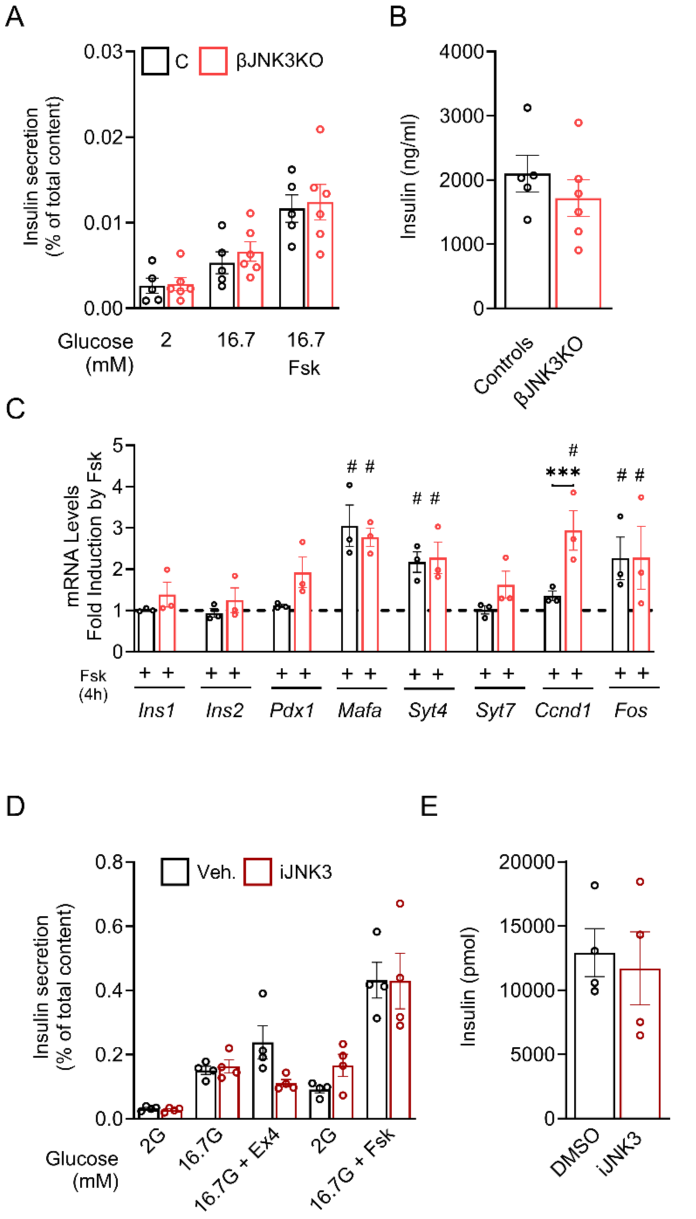

Supplemental Figure 8 (S8). Integrity of the cAMP/PKA/CREB axis downstream of GLP-1R activation using Forskolin. A) Control and βJNK3KO islets. B) Total insulin content from control and βJNK3KO islets. C) RT-PCR for β-cell identity and CREB-responsive genes in control and βJNK3KO islets treated with forskolin for 4 h. D) Human islets ± iJNK3 (4 replicates of donor#15). E) Total insulin content from human islets ± iJNK3. Data are expressed as means ± SEM. Statistical significance was determined by Two-way ANOVA. #P < 0.05 within the same group.

Figure S9

Bioinformatic prediction of the putative downstream transcription factors motifs that are activated by JNKs in the promoter region of Glp1 receptor gene

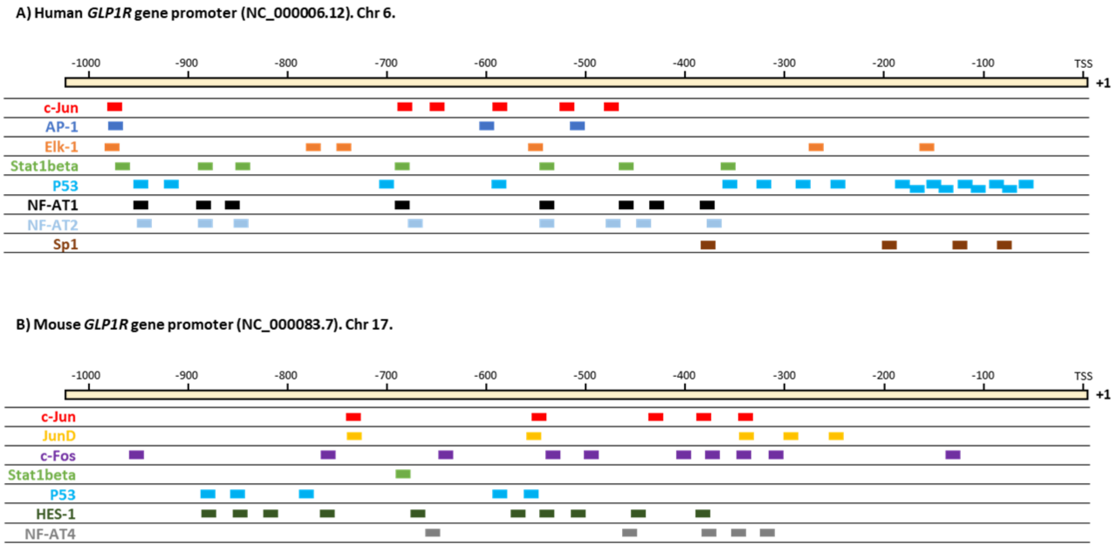

Supplemental Figure 9 (S9). Bioinformatic analysis of the promoter region of the *Glp1r* gene in human and mouse showed enrichment of putative binding sites for transcription factors targeted by JNKs, such as c-JUN, JUNB, Elk1, and other substrates

Figure S10

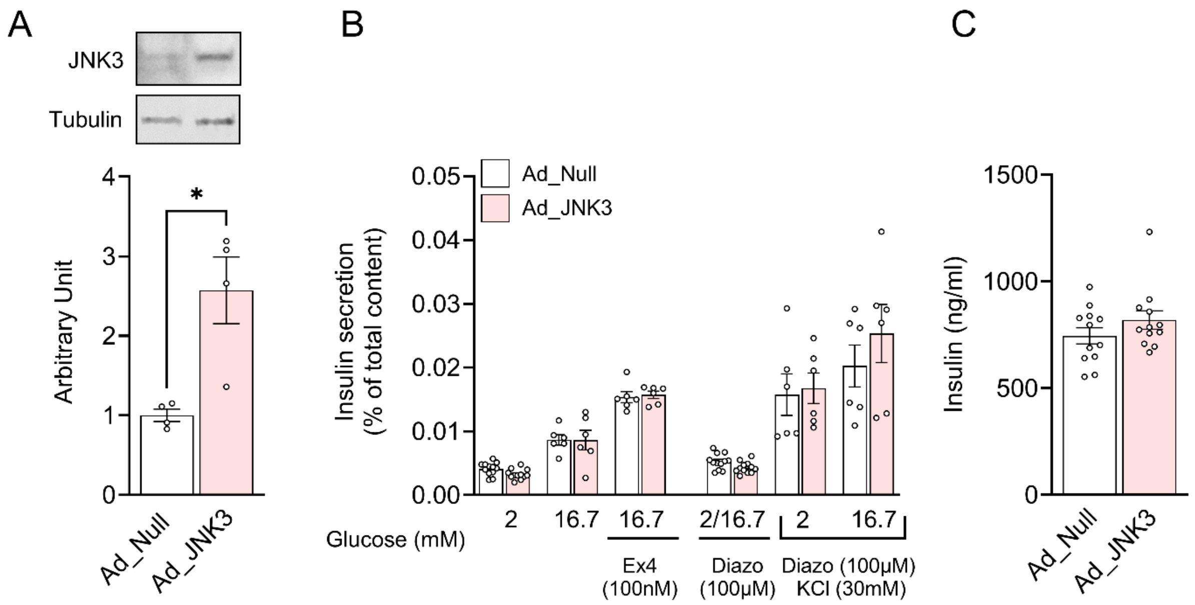

Supplemental Figure 10 (S10). JNK3 overexpression in mouse islets using adenovirus. A) JNK3 expression. B) GSIS alone or with Exendin-4 in Ad_Null vs. Ad_JNK3–infected islets. Data are expressed as means ± SEM. Statistical significance was determined by two-way ANOVA. *P < 0.05 between groups.

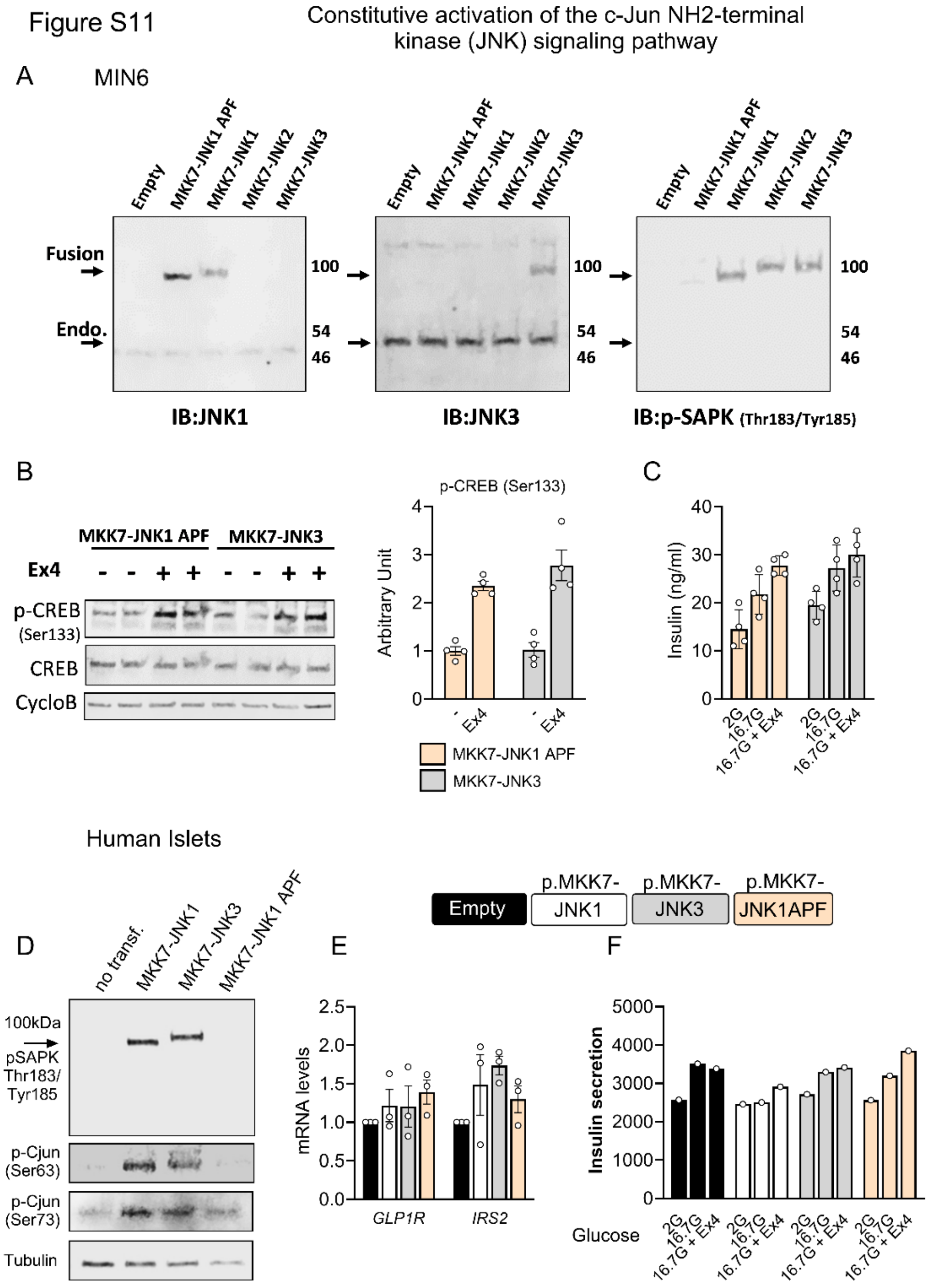

Supplemental Figure 11 (S11). Overactivation of JNKs. The MKK7-JNK fusion proteins contain residues 1–443 of MKK7 fused to JNK1α1 (1–383), JNK2α2 (1–423), or JNK3α2 (1–463). Point mutations were introduced to generate phosphorylation-negative JNK1 (Thr180–Pro–Tyr182 → Ala–Pro–Phe), preventing phosphorylation of JNK1 when fused with constitutively active MKK7 (MKK7-JNK1APF). A) Transient transfection of MIN6 cells with each construct. B) CREB phosphorylation in MIN6 cells after 1 h of Exendin-4 treatment. C) GSIS alone or with Exendin-4. D) Phosphorylation state of JNK assessed by anti–phospho-SAPK immunoblot. E) RT-PCR for *GLP1R* and *IRS2* in human islets transiently transfected with the constructs. F) GSIS alone or with Exendin-4 in human islets (donors #7-8, 9, 15).

Figure S12

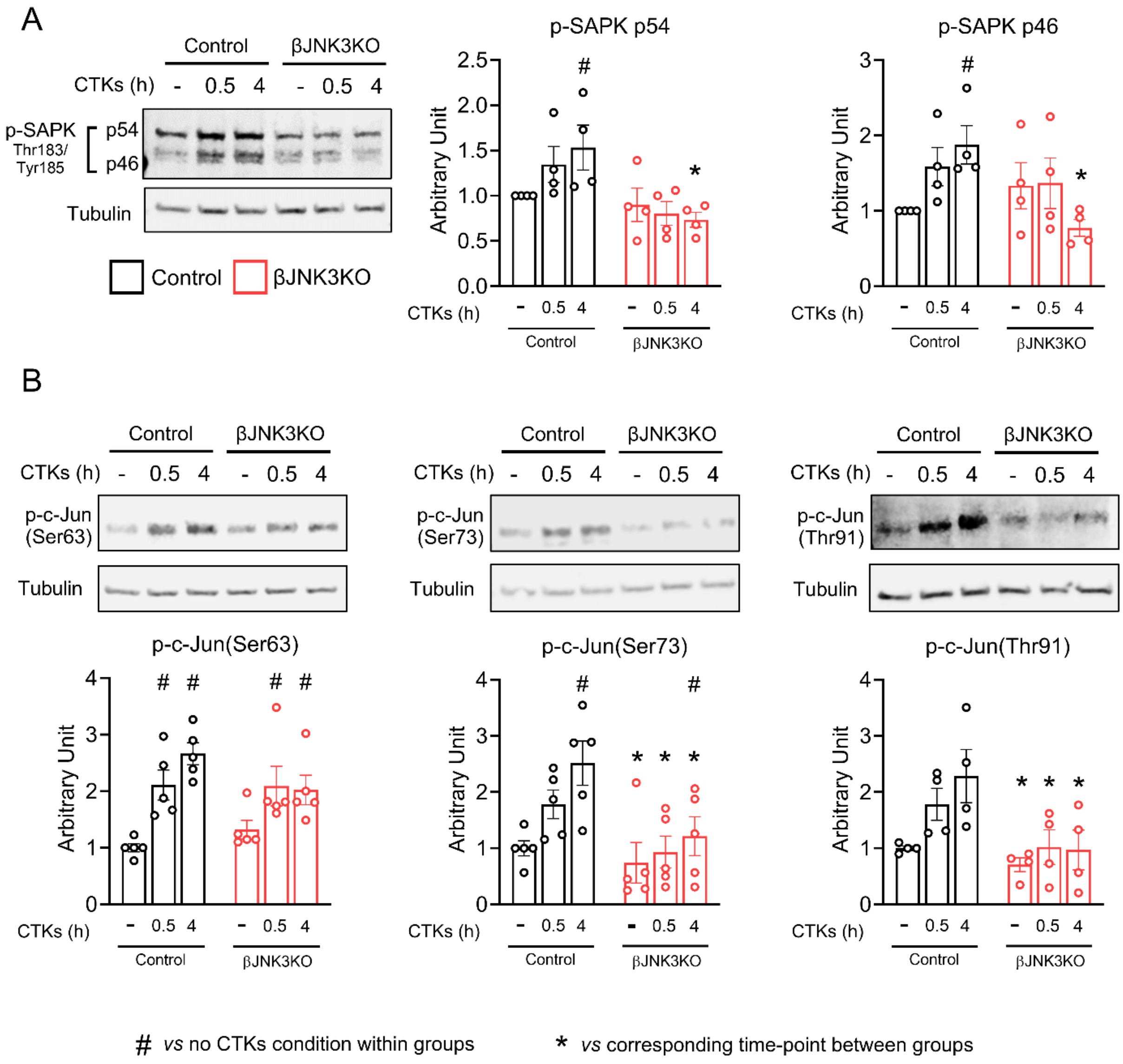

Supplemental Figure 12 (S12). Effects of CTKs on stress-activated protein kinases (SAPKs) and c-Jun phosphorylation are reduced in βJNK3KO islets. A) phosphorylation of SAPK (JNKs) at Threonine 183 and Tyrosine 185 and B) phosphorylation of c-Jun at Serine 63, Serine 73 and Threonine 91 were assessed by immunoblotting of isolated islets from 4-6 months old Control and βJNK3KO male and female mice treated with proinflammatory cytokines for 0.5 and 4 hours. #$P < 0.05$ compared to no CTKs condition within groups and *$P < 0.05$ compared to corresponding time-point between groups. The results are expressed as means ± SEM.

Table S1. Checklist for Reporting Human Islet Preparations Used in Research

Islets Preparation	Donor 1	Donor 2	Donor 3	Donor 4	Donor 5	Donor 6	Donor 7
Unique identifier	HP-23093-01	HP-23098-01	HP-23166-01	HP-23199-01	HP-23200-01	HP-23207-01	HP-23293-01
Donor age (years)	63	42	50	63	57	41	44
Donor sex (M/F)	F	F	F	M	M	M	F
Donor BMI (Kg/m^2)	22.1	29.3	28.8	21.3	30.8	33.8	24.1
Donor HbA1c or other measure of blood glucose control	5.60%	5.20%	5.60%	5.50%	5.20%	5.00%	5.40%
Origin/source of islets	Prodo Labs	Prodo Labs	Prodo Labs	Prodo Labs	Prodo Labs	Prodo Labs	Prodo Labs
Islet isolation centre	Prodo Labs	Prodo Labs	Prodo Labs	Prodo Labs	Prodo Labs	Prodo Labs	Prodo Labs
Donor history of diabetes? Yes/No	No	No	No	No	No	No	No
Donor cause of death	Stroke	Stroke	Stroke	Stroke	anoxic event	stroke	stroke
Estimated purity (%)	90	95	80-85	95	95	95	90
Estimated viability (%)	95	95	95	95	95	95	95
Total culture time (h)d	4-5 days	4-5 days	4-5 days	4-5 days	4-5 days	4-5 days	4-5 days
Functional measurement	GSIS	GSIS	GSIS	GSIS	GSIS	GSIS	GSIS

Islets Preparation	Donor 8	Donor 9	Donor 10	Donor 11	Donor 12	Donor 13	Donor 14	Donor 15
Unique identifier	HP-23301-01	HP-23305-01	HP-24284-01	HP-24298-01	HP-24303-01	HP-24312-01	HP-24340-01	nPOD 6630
Donor age (years)	64	47	55	61	49	66	55	27
Donor sex (M/F)	M	M	F	M	M	M	F	F
Donor BMI (Kg/m^2)	25.5	28.3	23.3	29.5	24.5	33.7	29.7	24.4
Donor HbA1c or other measure of blood glucose control	5.20%	5.30%	5.50%	5.70%	5.00%	5.90%	5.10%	5.00%
Origin/source of islets	Prodo Labs	Prodo Labs	Prodo Labs	Prodo Labs	Prodo Labs	Prodo Labs	Prodo Labs	nPOD
Islet isolation centre	Prodo Labs	Prodo Labs	Prodo Labs	Prodo Labs	Prodo Labs	Prodo Labs	Prodo Labs	nPOD
Donor history of diabetes? Yes/No	No	No	No	No	No	No	No	No
Donor cause of death	Stroke	Stroke	Stroke	Stroke	Stroke	Stroke	Stroke	Cardiac arrest
Estimated purity (%)	90	95	90	90	90	90	85	-
Estimated viability (%)	95	95	95	95	95	95	95	-
Total culture time (h)d	4-5 days	4-5 days	4-5 days	4-5 days	4-5 days	4-5 days	4-5 days	4-5 days
Functional measurement	GSIS	GSIS	GSIS	GSIS	GSIS	GSIS	GSIS	GSIS

Adapted from (49).

Table S2. Antibodies

**Antibody**	**Specie**	**Source (Catalog)**
Imunofluorescence		
INSULIN	Guinea Pig	Dako
GLP-1R	Mouse	DSHB (mAB 7F38)
Western Blot		
IRS2	Rabbit	Cell Signaling (4502)
JNK1	Mouse	Cell Signaling (3708)
JNK2	Rabbit	Cell Signaling (9258)
JNK3	Rabbit	Cell Signaling (2305)
pSAPK/JNK (Thr183/Tyr185)	Rabbit	Cell Signaling (4668)
pPKA Substrates (RRXS*/T*)	Rabbit	Cell Signaling (9624)
pCREB (Ser133)	Rabbit	Cell Signaling (9198)
CREB	Mouse	Cell Signaling (9104)
pC-JUN (Ser63)	Rabbit	Cell Signaling (91952)
pC-JUN (Ser73)	Rabbit	Cell Signaling (3270)
pC-JUN (Thr91)	Rabbit	Cell Signaling (2303)
pC-JUN (Thr93)	Rabbit	Cell Signaling (2993)
GLP-1R	Goat	OriGene (TA326758)
CASPASE 3	Rabbit	Cell Signaling (9662)
CLEAVED CASPASE 3	Rabbit	Cell Signaling (9664)
TUBULIN	Mouse	Thermofisher (T5168)
CYCLOPHILIN B	Rabbit	Thermofisher (PA1-027A)

Table S3. Primer Sequences

Genes Mice	Forward 3'-5'		Reverse 3'-5'
Mapk8	AACAGCTCGGAACACCTTGT		CTCTCGCCTGACTGGCTTTA
Mapk9	GACCAGCCTTCAGCACAGAT		GTGTGCTCAGTGGACATGGA
Mapk10	TGGGATCATCCACAGGGACT		CACGTTCTCCTTGTAGCCCA
Glp1r	GGGTCTCTGGCTACATAAGGACAAC		AAGGATGGCTGAAGCGATGAC
Ins1	GAAGTGGAGGACCCACAAGTG		CTGAAGGTCCCCGGGGCT
Ins2	ATGGCCCTGTGGATGCGCTT		CTAGTTGCAGTAGTTCTCCAGCTGG
Irs2	CACAATTCCAAGCGCCACAA		TGGTAGCGCTTCACTCTTTCA
Gck	CTGTTAGCAGGATGGCAGCTT		TTTCCTGGAGAGATGCTGTGG
Pdx1	CAGTGGGCAGGAGGTGCTTA		GGGCCGGGAGATGTATTTGTT
Ldha	ATGAAGGACTTGGCGGATGA		ATCTCGCCCTTGAGTTTGTCTT
Ldhb	GGGAAAGTCTCTGGCTGATGAA		CTGTCACAGAGTAATCTTTATCGGC
Mafa	CAAGGAGGAGGTCATCCGAC		TCTCCAGAATGTGCCGCTG
Slc2a2	ATTACCGACAGCCCATCCTC		AGCACAGAGACAGCCGTGAA
Syt4	CCGCGTGGAATTCGATGAAA		GACAGTGAAGACGAGGCCAA
Syt7	CGAAGGGGACCATGTACCG		TCTTGTAGCGTTTGCCCAGT
Ccnd11	TCAAGTGTGACCCGGACTG		ATGTCCACATCTCGCACGTC
Fos	TACTACCATTCCCCAGCCGA		CTGCGCAAAAGTCCTGTGTG
CycloB	GGAGATGGCACAGGAGGAA		GCCCGTAGTGCTTCAGCTT
18S	GCAATTATTCCCCATGAACG		GGGACTTAATCAACGCAAGC

Genes Human	Forward 3'-5'		Reverse 3'-5'
GLP1R	TGGATGTATAGCACAGCCGC		CCCCAGGGGACAACAAACAG
IRS2	GCCACCATCGTGAAAGAGTG		TGAAACAGTGCTGAGCGTCT
INS	GGACAGGCTGCATCAGAAGA		ATTGTTCCACAATGCCACGC
SLC2A2	GCCACACTCACACAAGACCT		AGGCCTGAAATTAGCCCACA
GCG	AAGAACTTGGCCGCAGACAT		CCCTGGCGGCAAGATTATCA
PDX1	TTGAGTTGGAGCACCCTGTG		GCAGTACTCCGAGCTGTCTC
SYT4	GTCCGGACTTTCAGATCCCT		TTGAACACTGCATTGGGGGT
SYT7	AGAGCACAGTGCAGCAGAAA		CTCCTGCAGGCAACCTCTTG
CCND1	GGACAGAATCCAGCCAGGAG		AAGACAAACTGGTGGGGCAA
FOS	ACACCCTCTGTCTGATCCCT		GCTGTTACACAGCGGTTTCC
PPIA	GCGTCTCCTTTGAGCTGTTTGCA		CCACCCTGACACATAAACCCTGGAA
