# Supplementary material for: JNK3 regulates β cell responses to incretins in human islets and mouse models
Source: J Clin Invest. 2026 Jan 2;136(1):e185707. doi: 10.1172/JCI185707 (PMC12721909; doi:10.1172/JCI185707)

Unedited blot and gel images

JNK3 regulates in vivo β-cell responses to incretins

Ruy A. Louzada[1 §], Marel Gonzalez Medina[1], Valentina Pita Grisanti[1], Jessica Bouviere[2], Amanda F. Neves[1], Joana Almaça[1], Myoung Sook Han[3], Roger Davis[3], Gil Leibowitz[4], Manuel Blandino-Rosano[1], and Ernesto Bernal-Mizrachi[1,5 §]

[1] Division of Endocrinology, Diabetes, and Metabolism, Department of Medicine, University of Miami Miller School of Medicine, Miami, FL
[2] Department of Biochemistry and Molecular Biology, University of Miami Miller School of Medicine, Miami, FL
[3] Program in Molecular Medicine, University of Massachusetts Chan Medical School, Worcester, MA, USA
[4] Diabetes Unit and Endocrine Service, Hadassah-Hebrew University Medical Center, Jerusalem, Israel.
[5] Veterans Affairs Medical Center, Miami, Florida, USA

Full unedited gel for Figure 1B

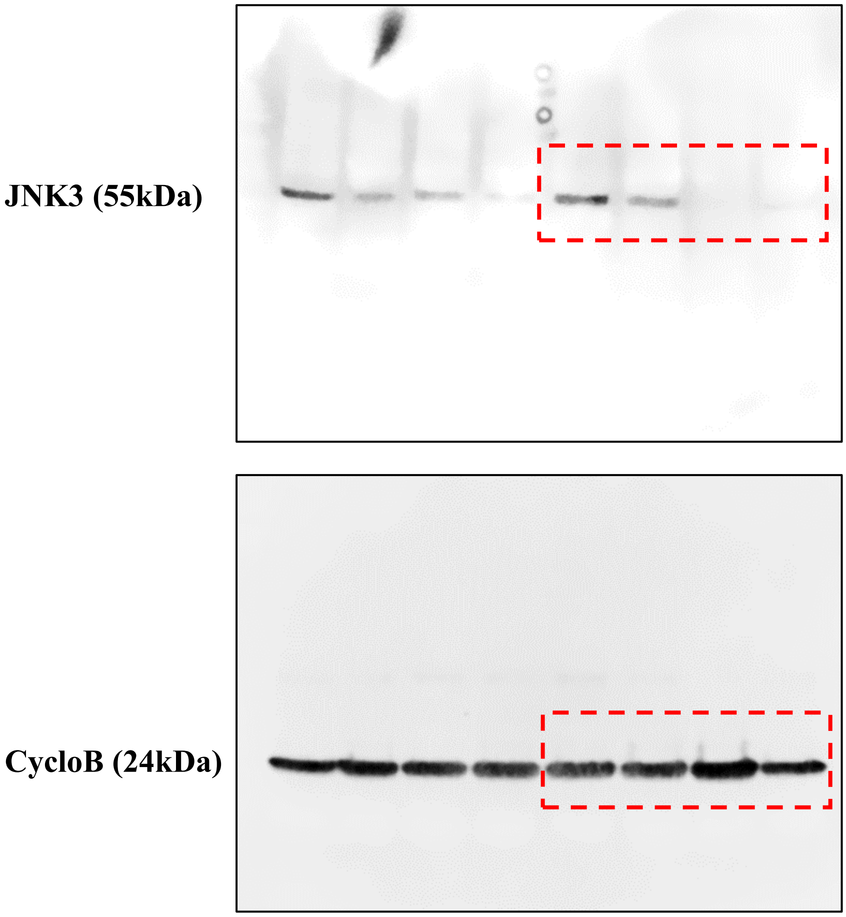

Full unedited gel for Figure 4A

P-CREB Ser133 (43kDa)

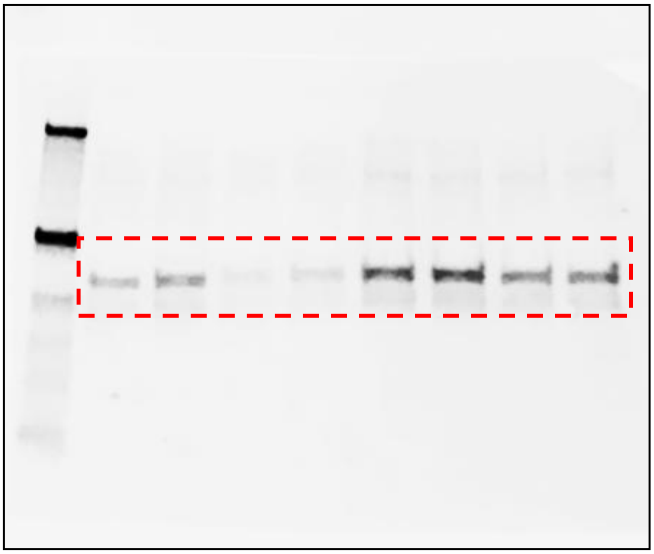

CycloB (24kDa)

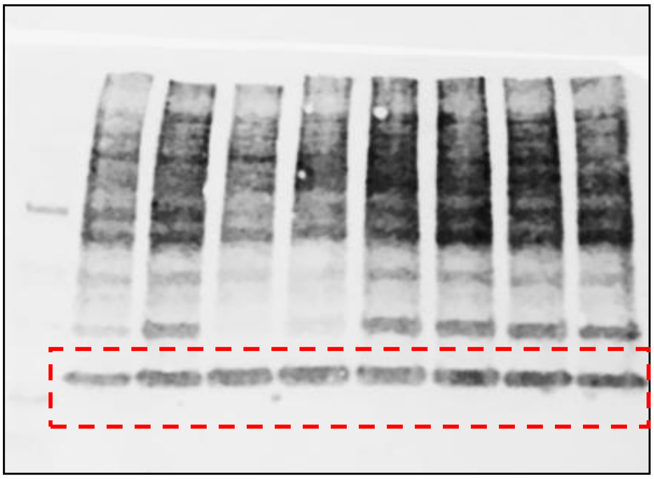

Full unedited gel for Figure 4B

P-CREB
Ser133
(43kDa)

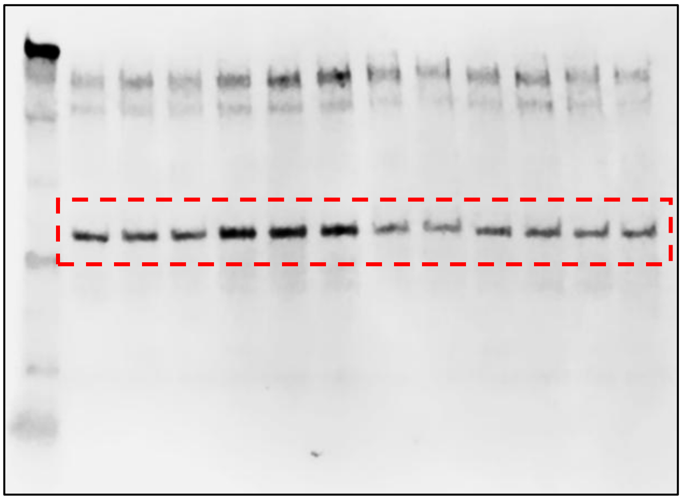

Tubulin
(52kDa)

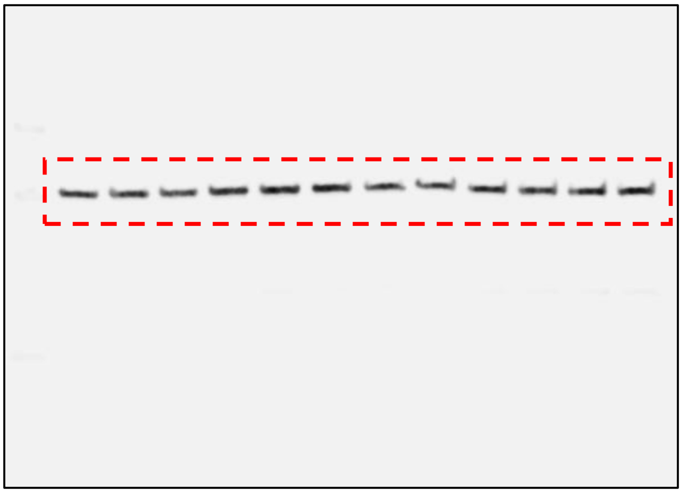

Full unedited gel for Figure 4C

P-CREB Ser133 (43kDa)

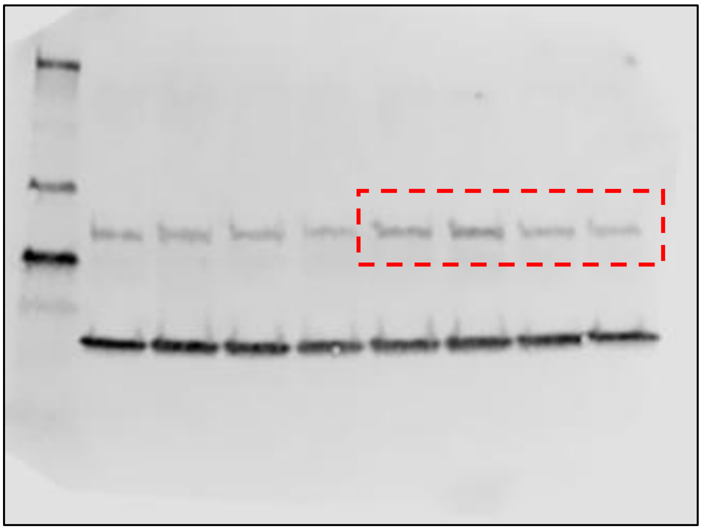

CycloB (24kDa)

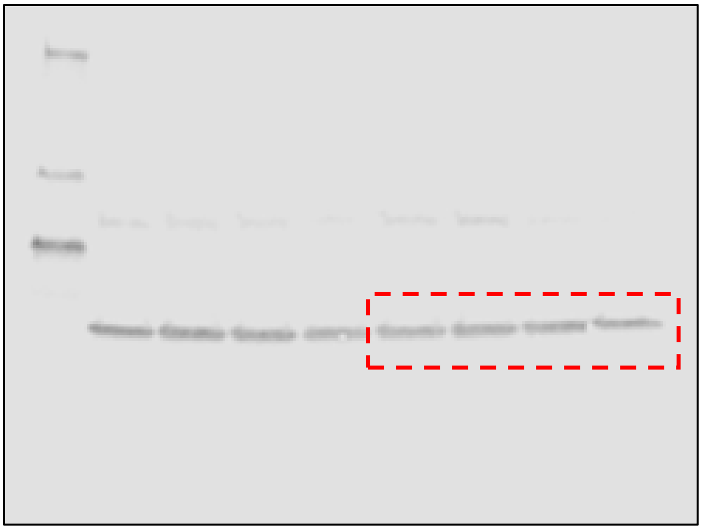

Full unedited gel for Figure 5E

GLP1R (54kDa)

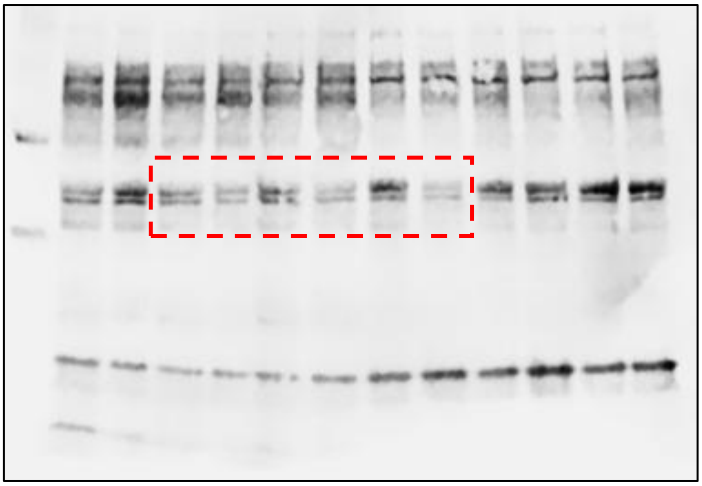

Tubulin (52kDa)

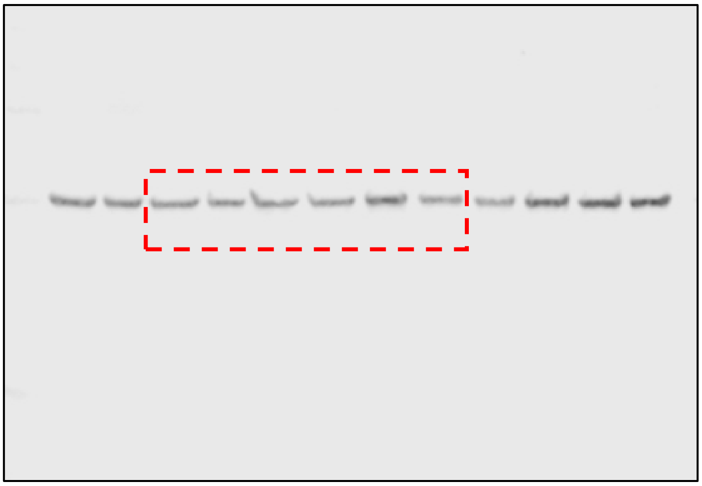

Full unedited gel for Figure 5F

pPKA Substrates RRXS*/T*

GLP1R (54kDa)

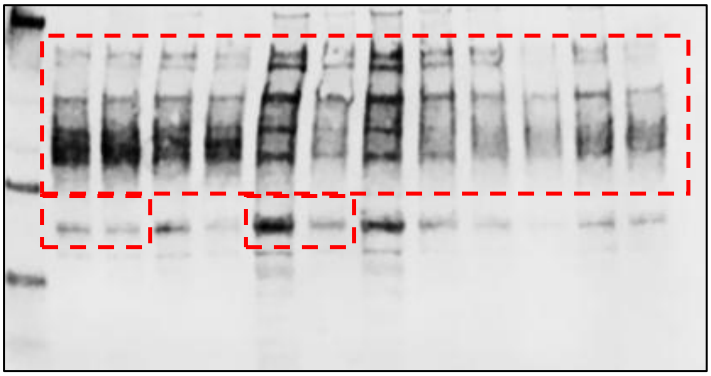

Tubulin (52kDa)

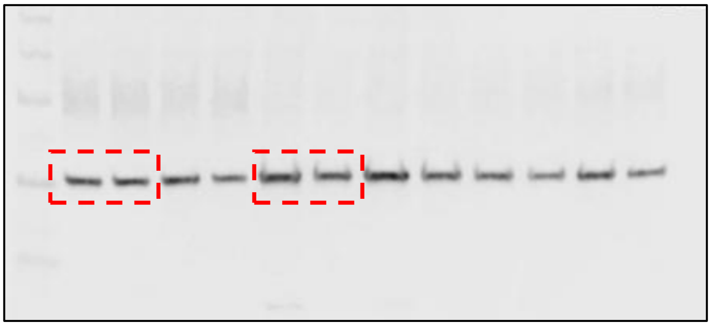

GLP1R (54kDa)

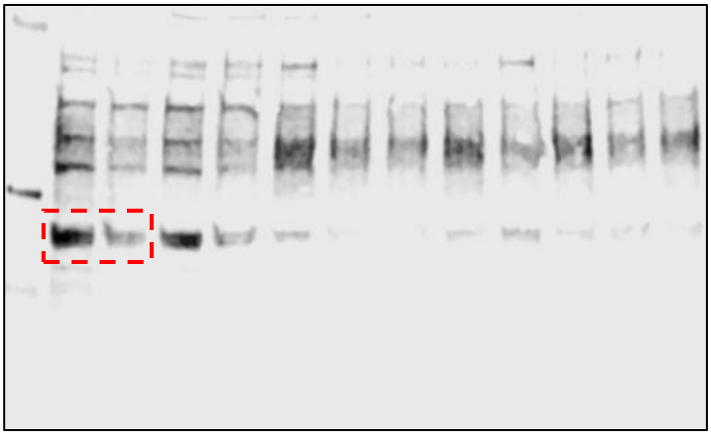

Tubulin (52kDa)

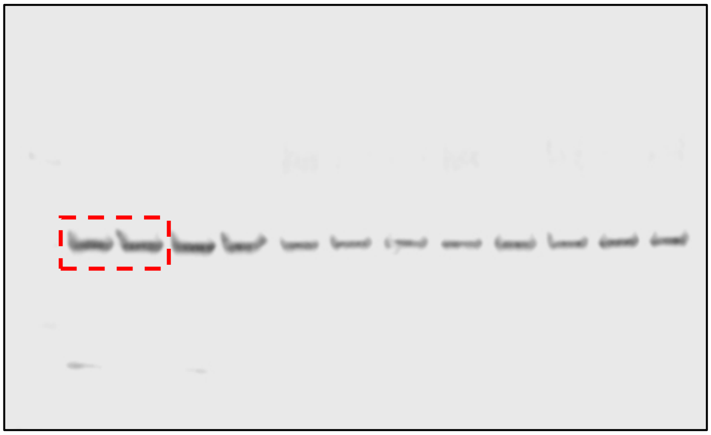

Full unedited gel for Figure 6B

p-SAPK
(100kDa)

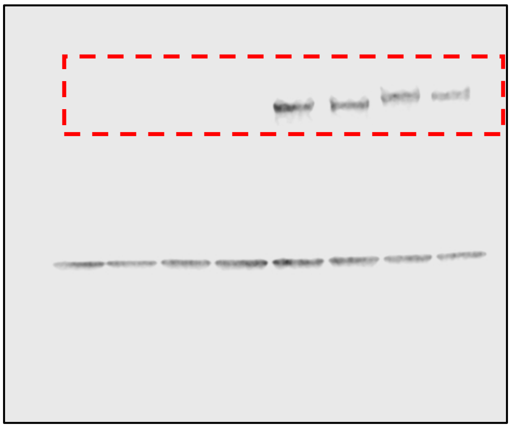

P-c-Jun
Thr 93
(48kDa)

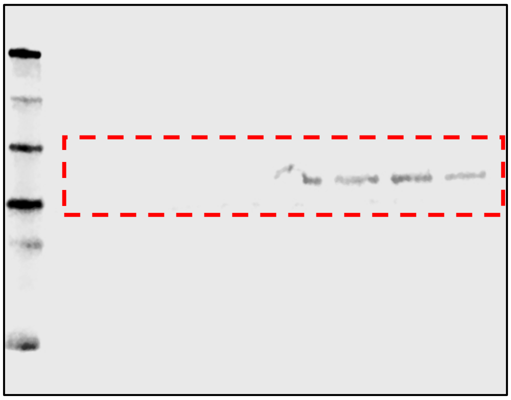

CycloB (24kDa)

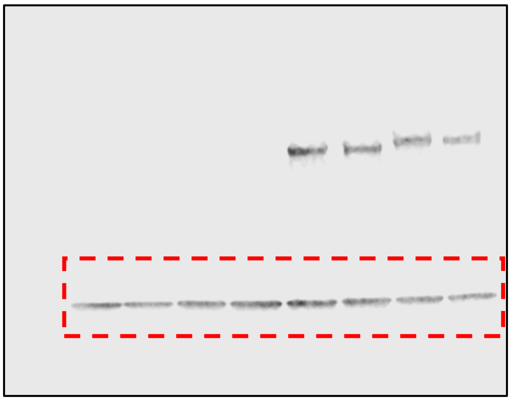

Full unedited gel for Figure 6B

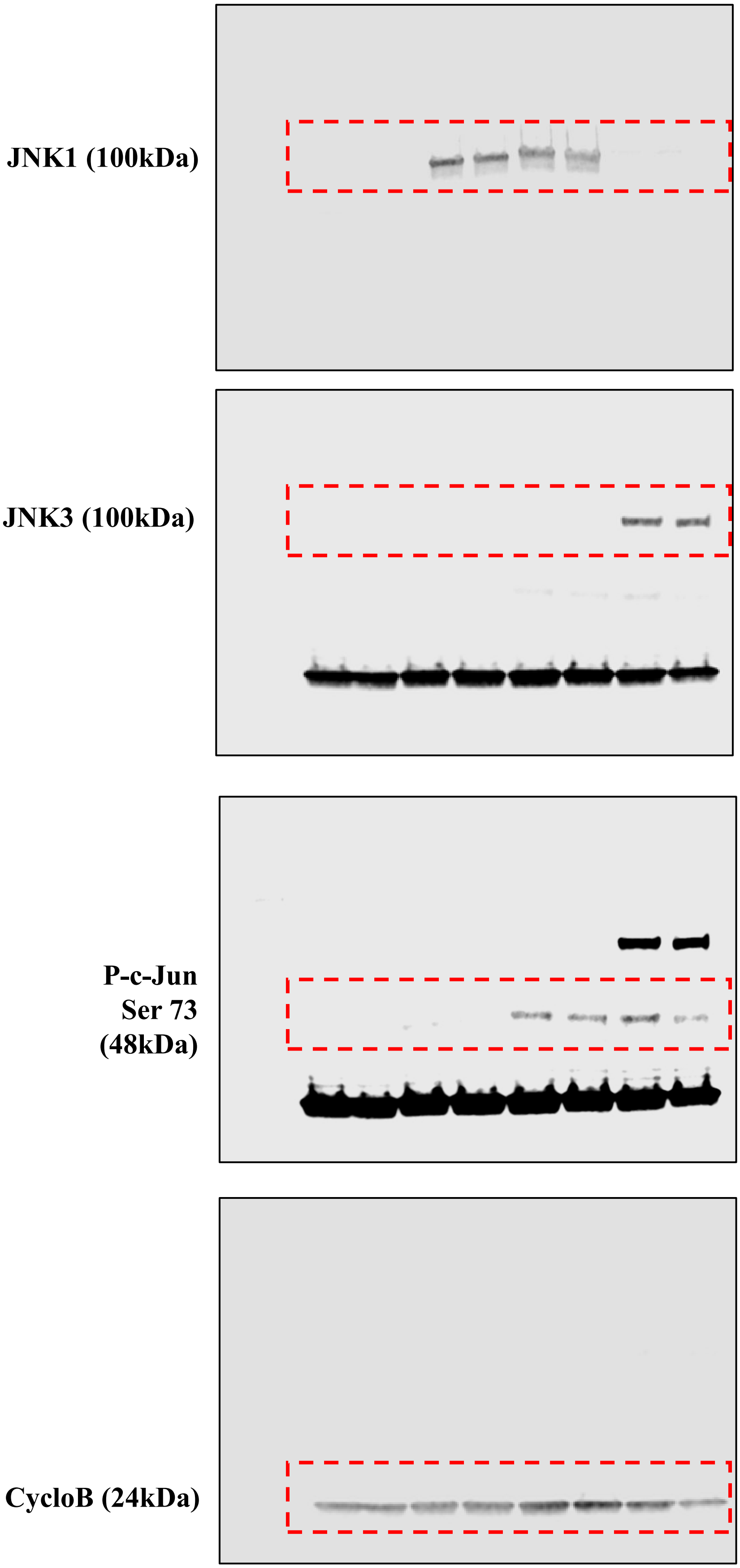

Full unedited gel for Figure 6B

P-c-Jun
Thr 91
(48kDa)

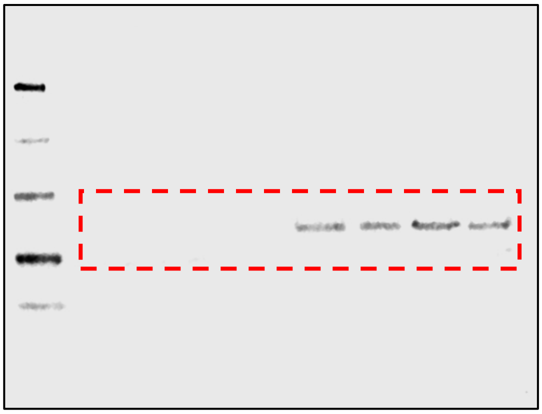

CycloB (24kDa)

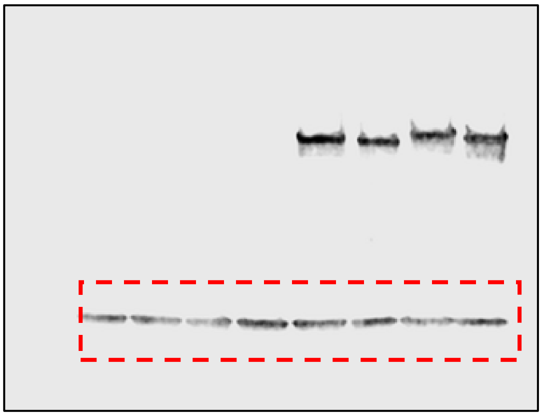

Full unedited gel for Figure 8A and S5A

IRS2 (185kDa)

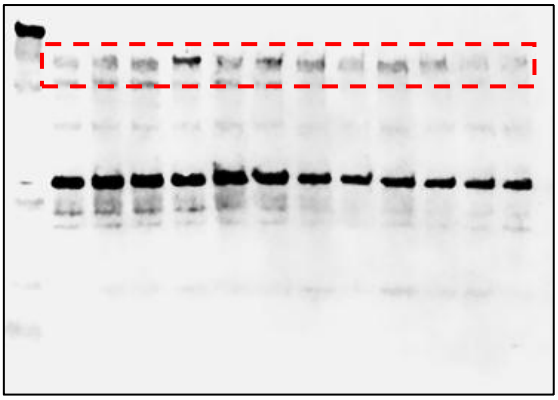

P-CREB Ser133 (43kDa)

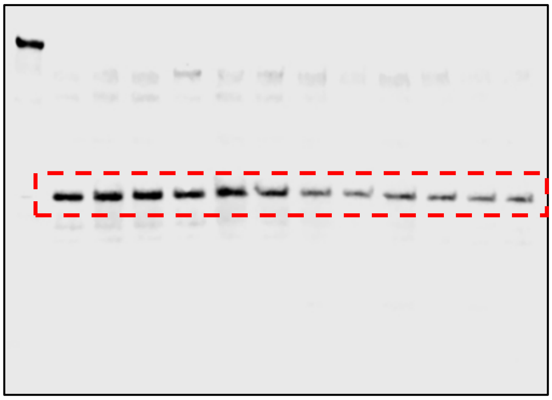

JNK3 (55kDa)

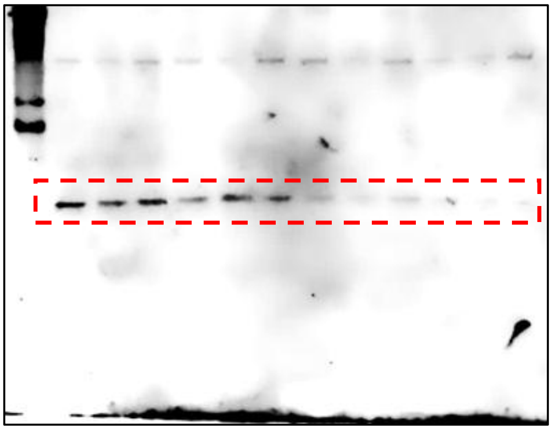

Tubulin (52kDa)

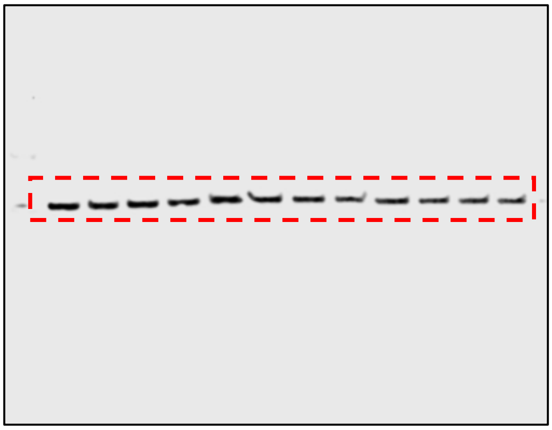

Full unedited gel for Figure 8B

IRS2 (185kDa)

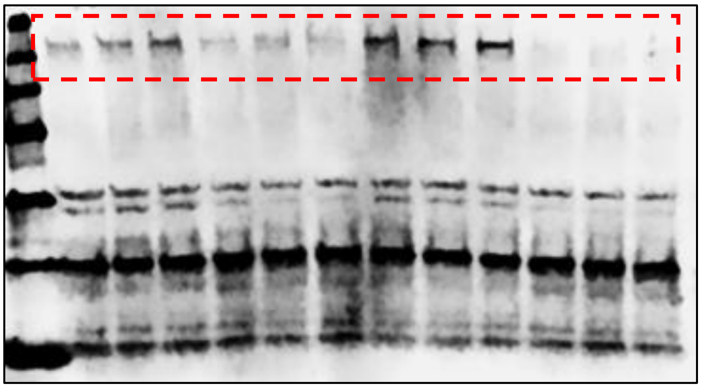

Caspase3(35kDa)

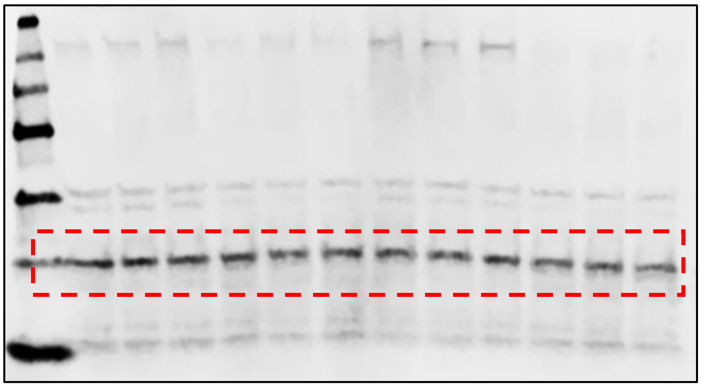

Cleaved Caspase3 (17kDa)

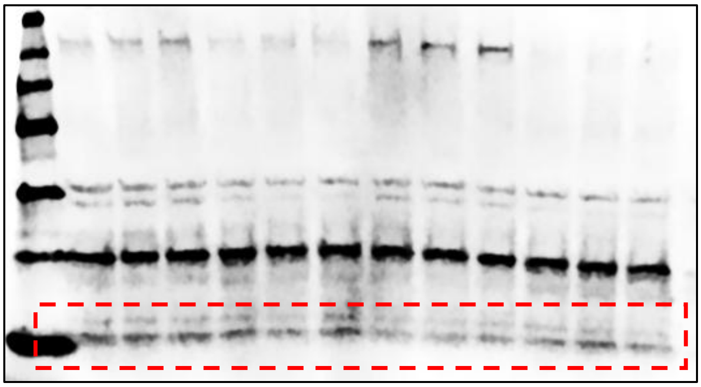

Tubulin (52kDa)

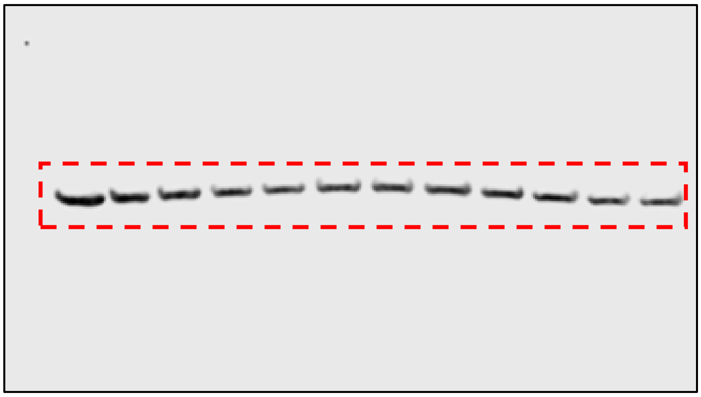

Full unedited gel for Figure 8D

IRS2 (185kDa)

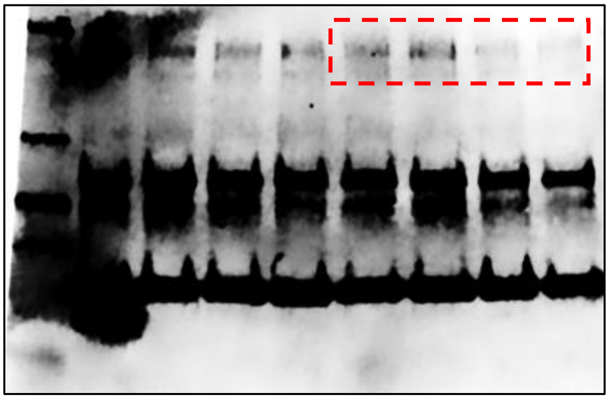

CycloB (24kDa)

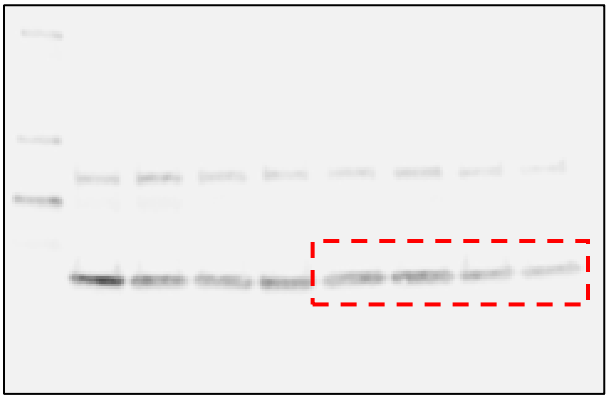

Full unedited gel for Figure S5B

P-c-Jun
Ser 63
(48kDa)

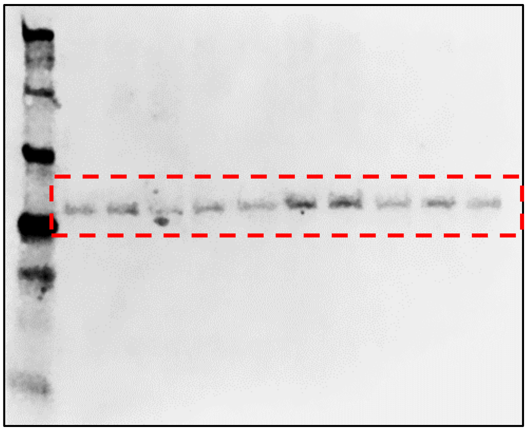

Tubulin
(52kDa)

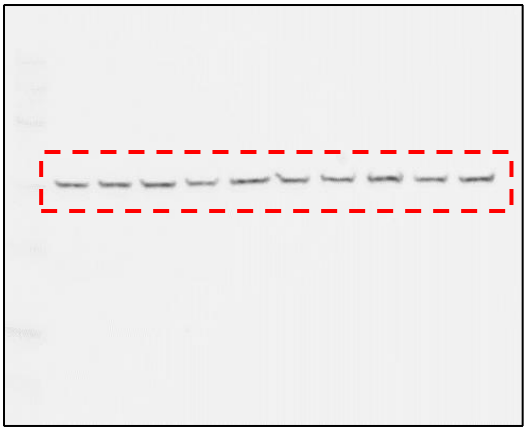

P-c-Jun
Ser 73
(48kDa)

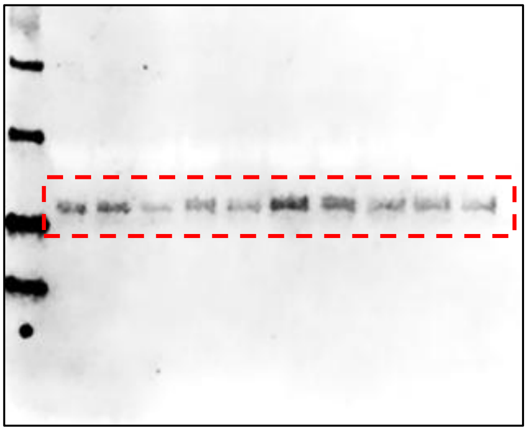

Tubulin
(52kDa)

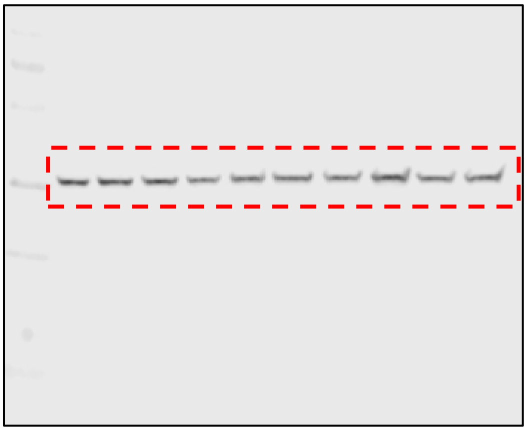

Full unedited gel for Figure S5C

IRS2 (185kDa)

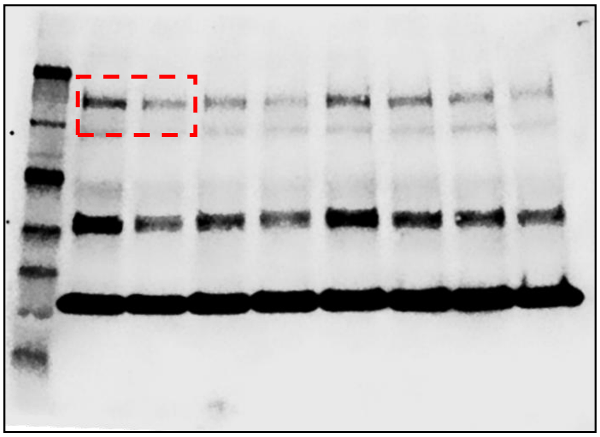

P-c-Jun Ser 73 (48kDa)

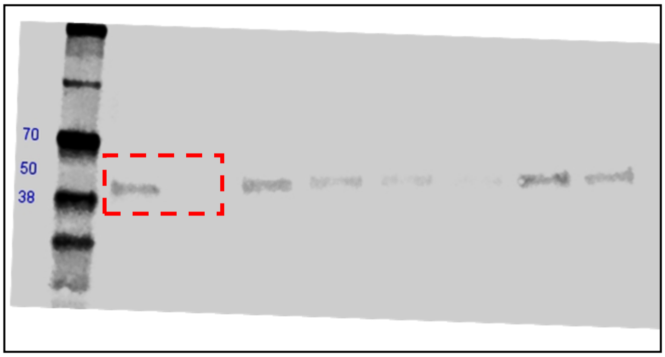

CycloB (24kDa)

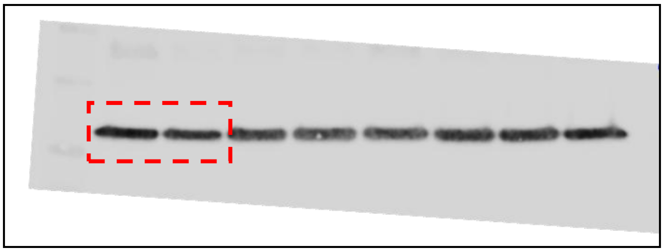

P-CREB Ser133 (43kDa)

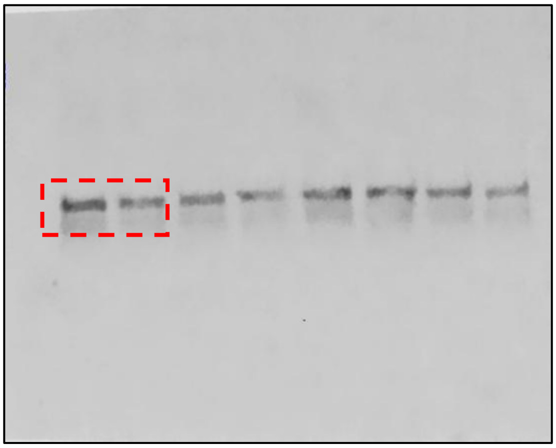

CycloB (24kDa)

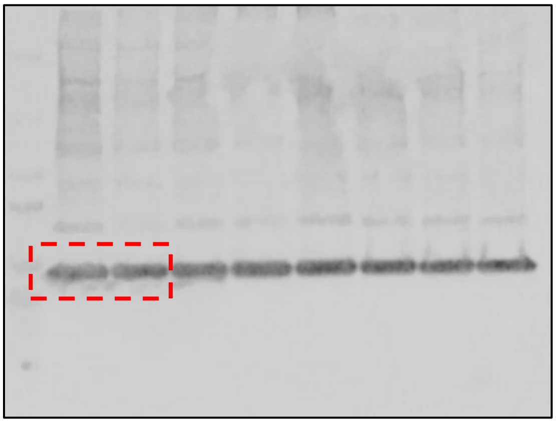

Full unedited gel for Figure S7A

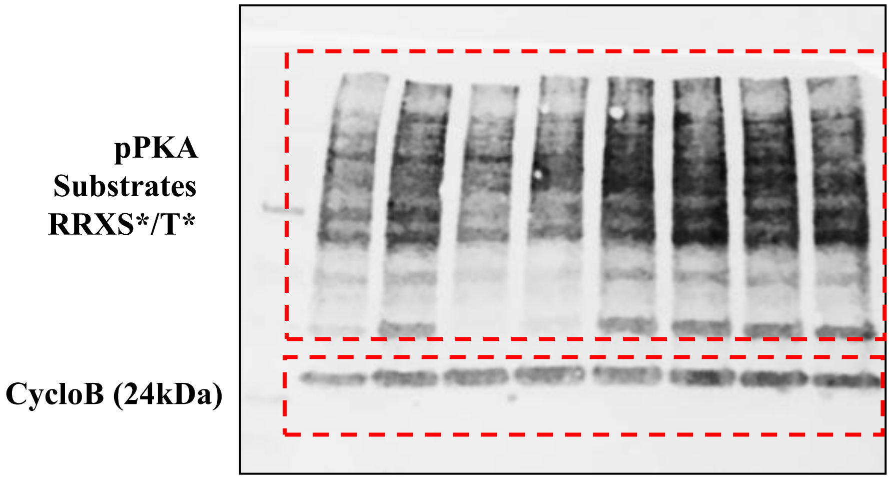

Full unedited gel for Figure S7B

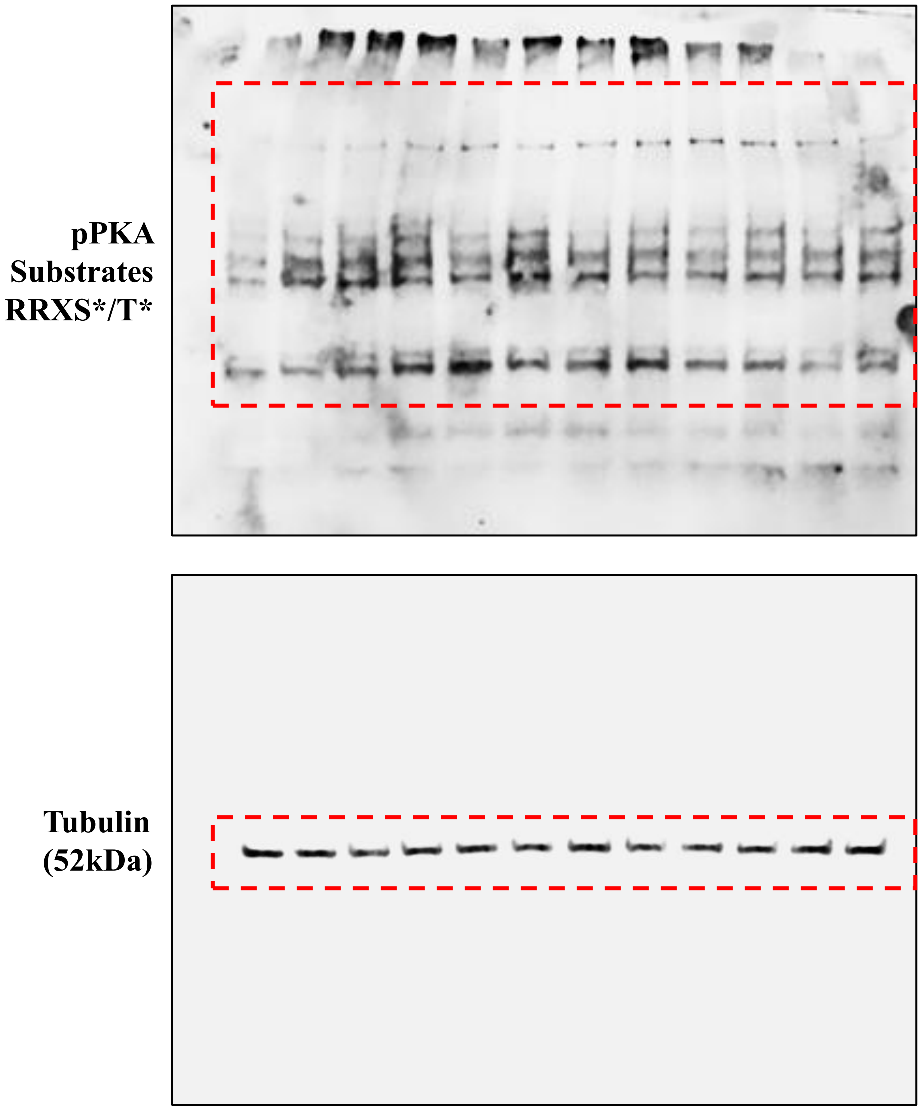

Full unedited gel for Figure S7C

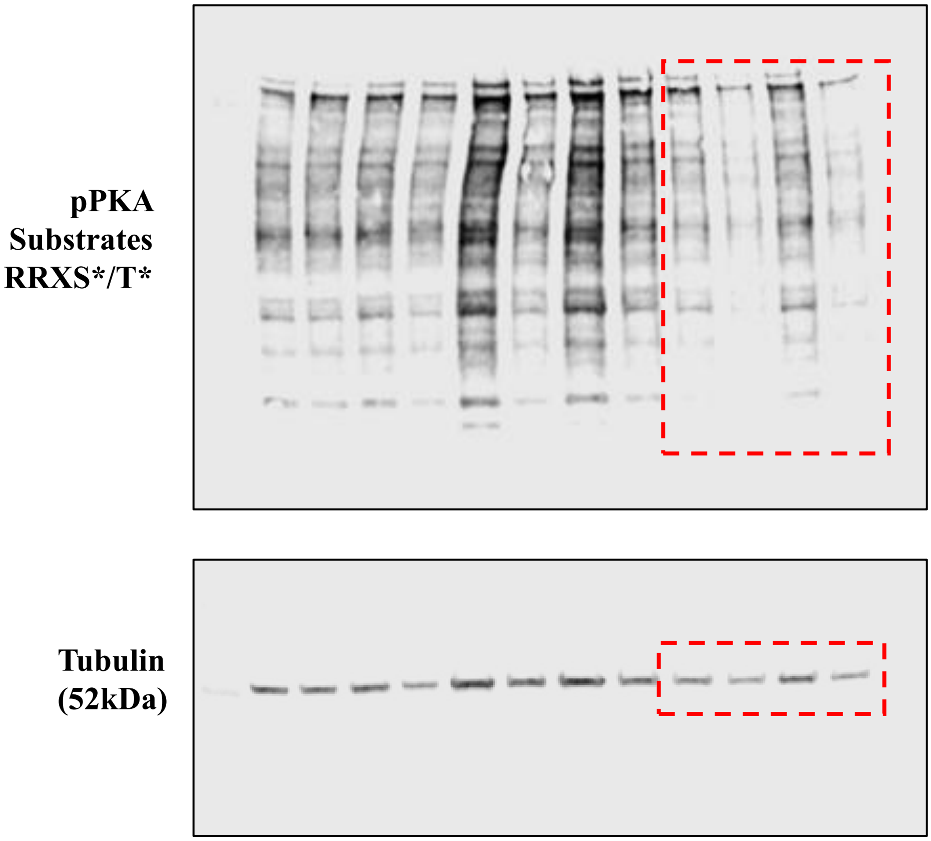

Full unedited gel for Figure S10

JNK3 (55kDa)

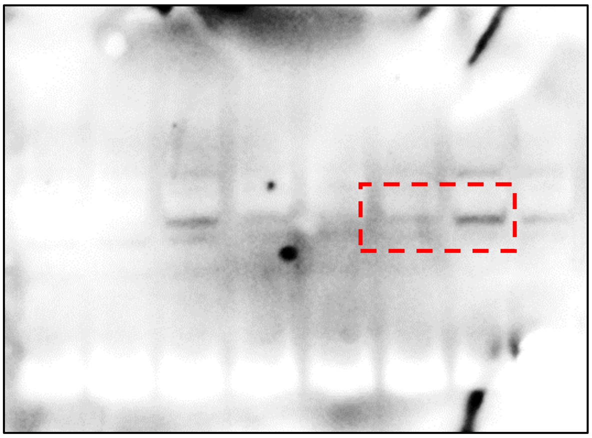

Tubulin (52kDa)

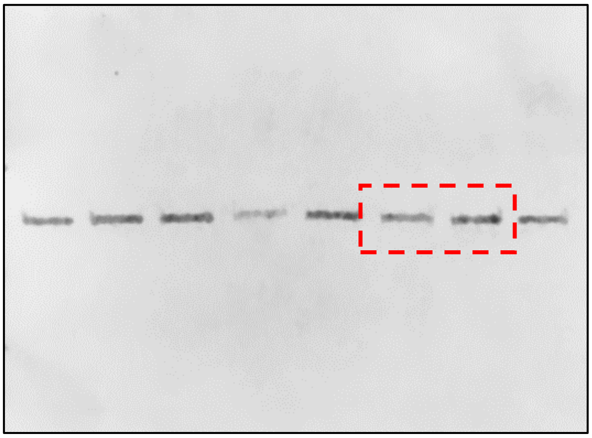

Full unedited gel for Figure S11A and B

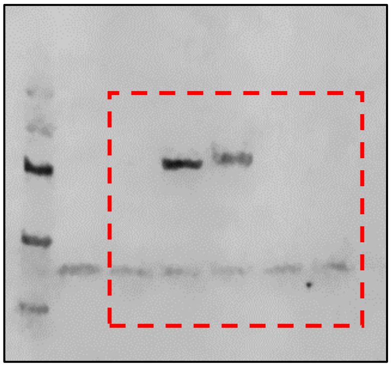

JNK1

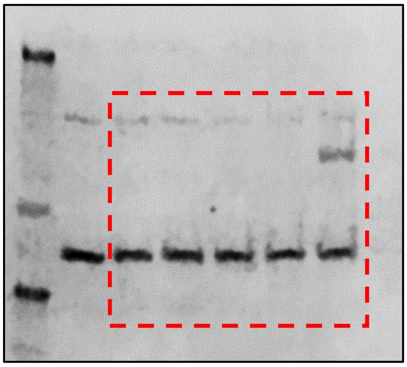

JNK3

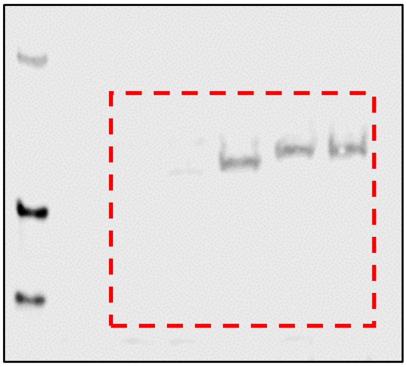

p-SAPK

P-CREB Ser133 (43kDa)

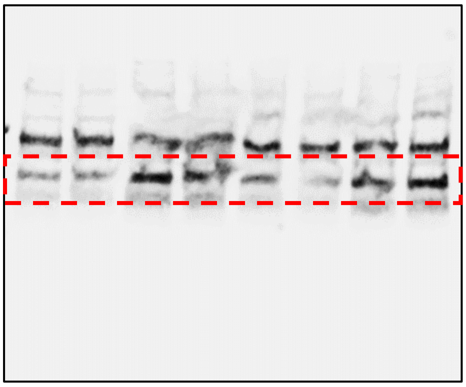

CREB (43kDa)

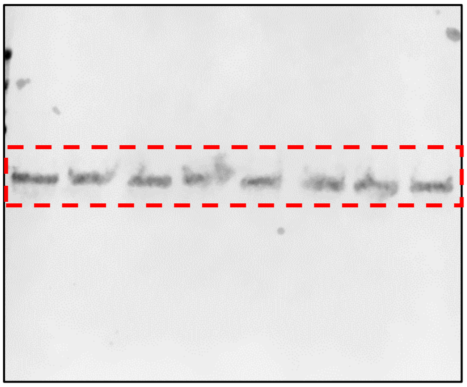

Tubulin (52kDa)

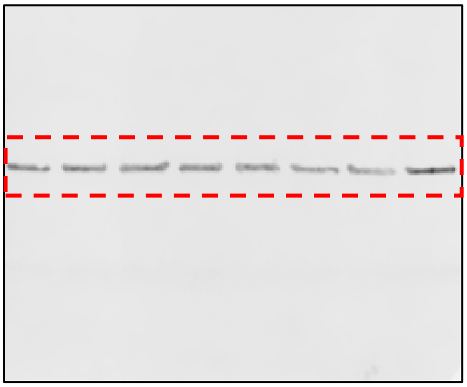

Full unedited gel for Figure S11E

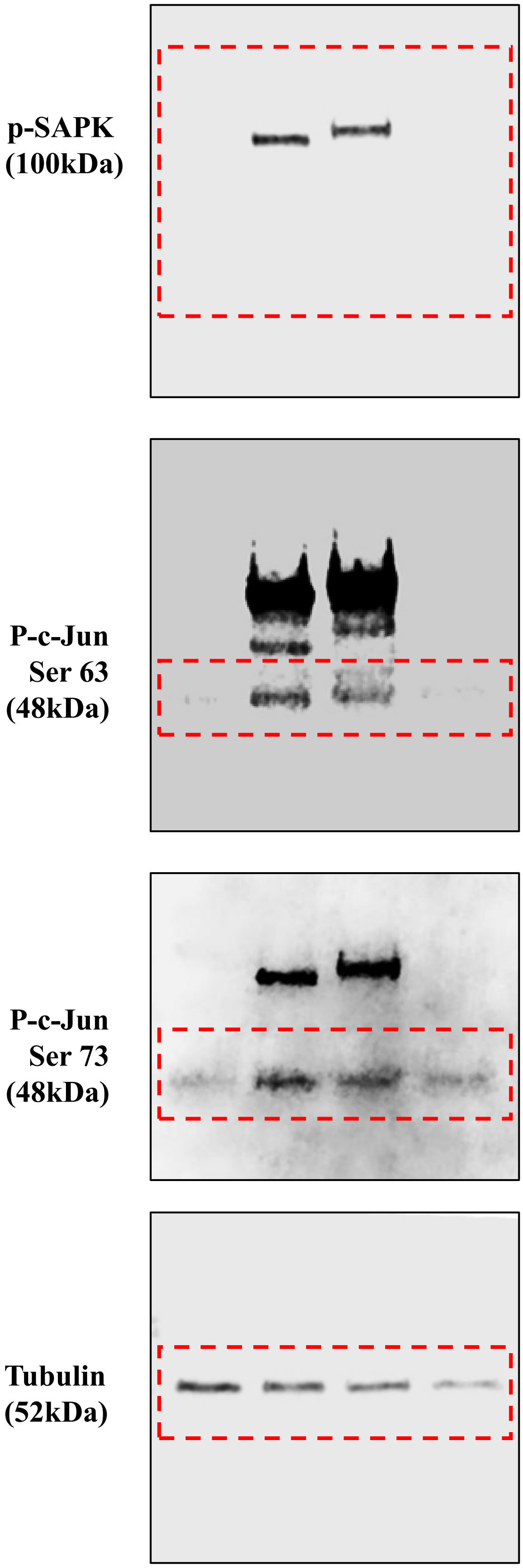

Full unedited gel for Figure S12A

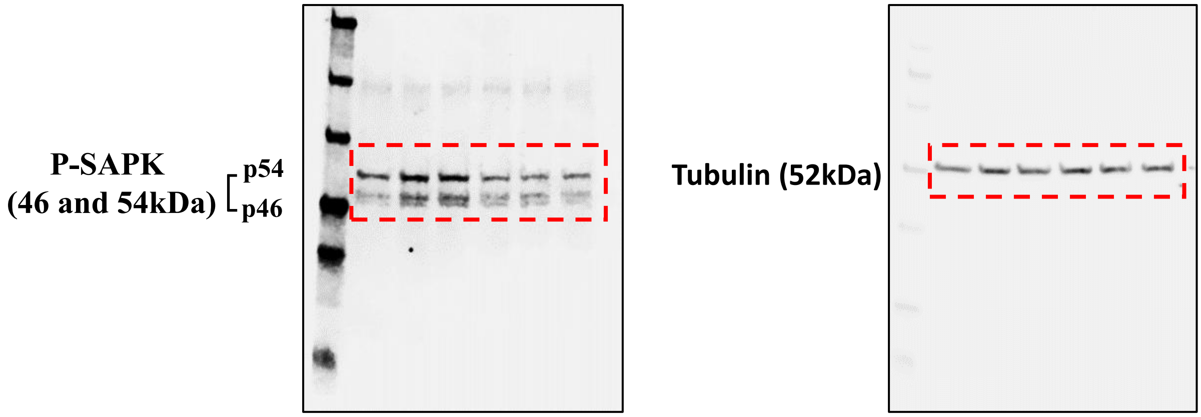

Full unedited gel for Figure S12B

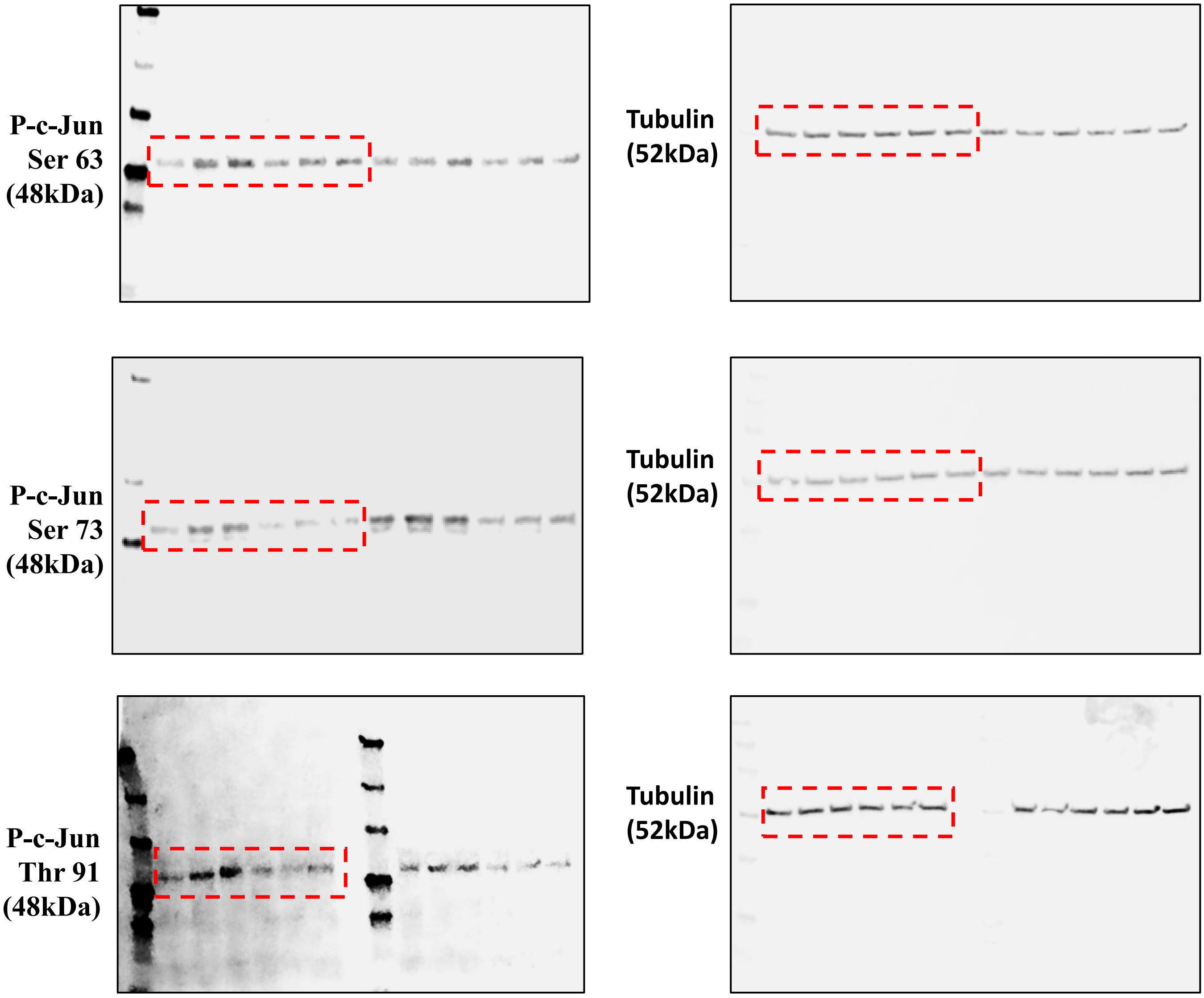

Supplement: Unedited blot and gel images [file jci-136-185707-s189.pdf]
